# A RecET-assisted CRISPR–Cas9 genome editing in *Corynebacterium glutamicum*

**DOI:** 10.1186/s12934-018-0910-2

**Published:** 2018-04-23

**Authors:** Bo Wang, Qitiao Hu, Yu Zhang, Ruilin Shi, Xin Chai, Zhe Liu, Xiuling Shang, Yun Zhang, Tingyi Wen

**Affiliations:** 1 CAS Key Laboratory of Pathogenic Microbiology and Immunology, Institute of Microbiology, Chinese Academy of Sciences, Beijing, 100101 China; 2 University of Chinese Academy of Sciences, Beijing, 100049 China; 3 Beijing Zhongke Eppen Biotech Co., Ltd, Beijing, 100085 China; 4 Savaid Medical School, University of Chinese Academy of Sciences, Beijing, 100049 China

**Keywords:** *Corynebacterium glutamicum*, CRISPR–Cas9, *recET*-encoded recombinase, Transformation efficiency, Genome editing, 1,2-Propanediol

## Abstract

**Background:**

Extensive modification of genome is an efficient manner to regulate the metabolic network for producing target metabolites or non-native products using *Corynebacterium glutamicum* as a cell factory. Genome editing approaches by means of homologous recombination and counter-selection markers are laborious and time consuming due to multiple round manipulations and low editing efficiencies. The current two-plasmid-based CRISPR–Cas9 editing methods generate false positives due to the potential instability of Cas9 on the plasmid, and require a high transformation efficiency for co-occurrence of two plasmids transformation.

**Results:**

Here, we developed a RecET-assisted CRISPR–Cas9 genome editing method using a chromosome-borne Cas9–RecET and a single plasmid harboring sgRNA and repair templates. The inducible expression of chromosomal RecET promoted the frequencies of homologous recombination, and increased the efficiency for gene deletion. Due to the high transformation efficiency of a single plasmid, this method enabled 10- and 20-kb region deletion, 2.5-, 5.7- and 7.5-kb expression cassette insertion and precise site-specific mutation, suggesting a versatility of this method. Deletion of *argR* and *farR* regulators as well as site-directed mutation of *argB* and *pgi* genes generated the mutant capable of accumulating l-arginine, indicating the stability of chromosome-borne Cas9 for iterative genome editing. Using this method, the model-predicted target genes were modified to redirect metabolic flux towards 1,2-propanediol biosynthetic pathway. The final engineered strain produced 6.75 ± 0.46 g/L of 1,2-propanediol that is the highest titer reported in *C. glutamicum*. Furthermore, this method is available for *Corynebacterium pekinense* 1.563, suggesting its universal applicability in other *Corynebacterium* species.

**Conclusions:**

The RecET-assisted CRISPR–Cas9 genome editing method will facilitate engineering of metabolic networks for the synthesis of interested bio-based products from renewable biomass using *Corynebacterium* species as cell factories.

**Electronic supplementary material:**

The online version of this article (10.1186/s12934-018-0910-2) contains supplementary material, which is available to authorized users.

## Background

*Corynebacterium glutamicum* is an important platform organism for modern biotechnology to produce amino acids, organic acids, nucleic acids, and bio-based products from the cheap and renewable biomass, such as glucose, sucrose, and xylose [1–3]. Genome editing to modify chromosome in way of deletion, integration or replacement is an efficient manner to metabolic engineering of this bacterium for overproducing the desired products, which is preferred than the traditional random mutagenesis [4]. Systems metabolic engineering guided by rational design using the genome-scale metabolic model requires a great deal of genetic modifications in *C. glutamicum* genome, which are laborious and time-consuming. Therefore, it is necessary to develop an easy and efficient genome editing method with extensive applicability to facilitate modifying the metabolic network of *C. glutamicum* [4–6].

The traditional approaches dependent on two rounds of homologous recombination require a non-replicating vector with a counter-selectable marker to screen target mutant in *C. glutamicum* ATCC 13032 [7]. The selection markers and vector elements need to be removed via a single crossover event, possibly resulting in undesired restoration of the wild type allele even in the presence of selective pressure. The counter selection by *sacB* gene leads to a 20–40% false-positive rate due to the spontaneous inactivation of SacB [8]. As another counter-selectable marker, *upp* encoding a uracil phosphoribosyltransferase is able to decrease the false-positive rate when it is concerted with I-*Sce*I [8]. A toxin counter-selectable cassette regulated by an antitoxin switch (TCCRAS) system can achieve 100% counter-selectable efficiencies for gene knockout and replacement [9]. To increase the marker eviction efficiency, Cre/mutant *loxP* system composed of *cre*-encoding recombinase and *loxP* sites are utilized to mediate reciprocal site-specific recombination between the *lox71* and *lox66* to facilitate easy removal of counter-selectable markers and undesirable vector elements [10, 11]. In spite of high counter-selectable efficiencies, these approaches are still inefficient due to their relatively low frequencies at which homologous recombination take place. To improve homologous recombination efficiency for gene deletion, the exonuclease-recombinase RecE and RecT (RecET) from the Rac prophage of *Escherichia coli* are expressed on a plasmid in *C. glutamicum* to mediate a double-crossover event via a dsDNA fragment containing the counter-selectable markers and homology arms [8, 12]. With the assistance of RecET expression, a linear dsDNA is resected by RecE to expose a 3-ended single-stranded DNA (ssDNA) tail for the binding of RecT, which contributes to promote the annealing of complementary DNA strand, strand exchange and strand invasion [8, 12]. The RecET-Cre/*loxP* system constructed in *C. glutamicum* ATCC 14067 only requires one round of recombination to obtain the target mutant [12].

The clustered regularly interspaced short palindromic repeat (CRISPR) as a bacterial immune defense system has been developed for genome editing in a wide range of organisms, such as bacteria and eukaryotes [13–17]. The type II CRISPR–Cas9 system from *Streptococcus pyogenes* is widely used for genome editing due to the inherent simplicity and flexibility in sequence requirements for sgRNA [13–15]. Cas9 from *S. pyogenes* (SpCas9) requires a single guide RNA (sgRNA) containing 20 bp complementary sequence of the target region and the SpCas9 binding scaffold to form sgRNA-SpCas9 ribonucleoprotein complex, which generates a double-strand break (DSB) at 20 bp-designated target region after recognizing a protospacer adjacent motif (PAM) sequence NGG [13]. DSB by Cas9 cleavage in the chromosome is lethal to many bacteria deficient in the endogenous nonhomologous end joining (NHEJ) mechanism, when DSB is unable be repaired through homologous recombination (HR) [13]. Cas9-mediated killing can help select mutations introduced through HR using a donor DNA as the repair template and eliminate cells carrying the wild-type genotype [16, 17]. So far, CRISPR–Cas9 system has been developed as a powerful tool to genome editing in many bacteria [18–24].

In recent studies regarding CRISPR–Cas9 assisted genome editing of *C. glutamicum*, several attempts to transform the plasmid harboring the native codon-encoded SpCas9 under different inducible promoters failed to obtain any colony, which might be attributed to a negative effect of its strong expression on cell viability [25, 26]. To decrease the translation level, attempts to utilize the codon-optimized *cas9* gene and different ribosomal binding sequences succeeded to express plasmid-borne Cas9 protein [26–28]. Two plasmids-based CRISPR–Cas9 editing system consisted of inducible expression cassette of Cas9 in one plasmid and sgRNA expression cassette together with a repair template in the other plasmid [27, 28]. However, the transformation with two plasmids harboring CRIPSR–Cas9 system yielded the abnormally large colonies in which their *cas9* genes were deleted from pCas9 plasmid, indicating the instability of pCas9. Furthermore, the manipulation procedure is relatively complicated due to the construction of two plasmids harboring genetic elements and transformation efficiency is low for the co-occurrence of the introduction of Cas9-sgRNA and repair template [double-stranded or single-stranded DNA (ssDNA)], resulting in a high requirement for transformation efficiency. The plasmid-borne dsDNA template can directly repair the DSB for gene deletion via HR in NHEJ-deficient *C. glutamicum*; however, it is necessary to express recombinase RecT to incorporate ssDNA to repair DSB [26], indicating that HR is a key factor in CRISPR–Cas9 editing system. Compared to Cas9, Cpf1 that utilizes a T-rich PAM site has less editing targets in the GC-rich *C. glutamicum* genome and only reaches 5–15% of editing efficiencies for gene deletion and insertion [25]. These editing methods have disadvantage in modifying endogenous genes or integrating heterologous genes to construct synthetic pathways. Hence, it is of great importance to develop an easy and efficient CRISPR–Cas9 system to implement an extensive genome editing including gene deletion, integration and mutation in *C. glutamicum* for academic studies and industrial applications.

In this study, to simplify the editing elements construction and minimize the physiological impacts of Cas9 on cells, we developed a CRISPR–Cas9 genome editing method in *C. glutamicum* by integrating *cas9* into chromosome and constructing a single plasmid harboring a sgRNA and repair DNA templates. Through introducing *recET*-encoded recombinase to increase the probability of homologous recombination, this genome editing method enables the deletion and integration of large DNA fragments, and accomplishes the precise site-directed mutations in the chromosome at a high efficiency. It was successfully applied to modify multiple genes for increasing 1,2-propanediol production. This developed genome editing method will facilitate engineering of native metabolic network or introducing novel pathways in *C. glutamicum* to synthesize the desired bio-based products.

## Results

### Optimization of CRISPR–Cas9 system

**Fig. 1:**
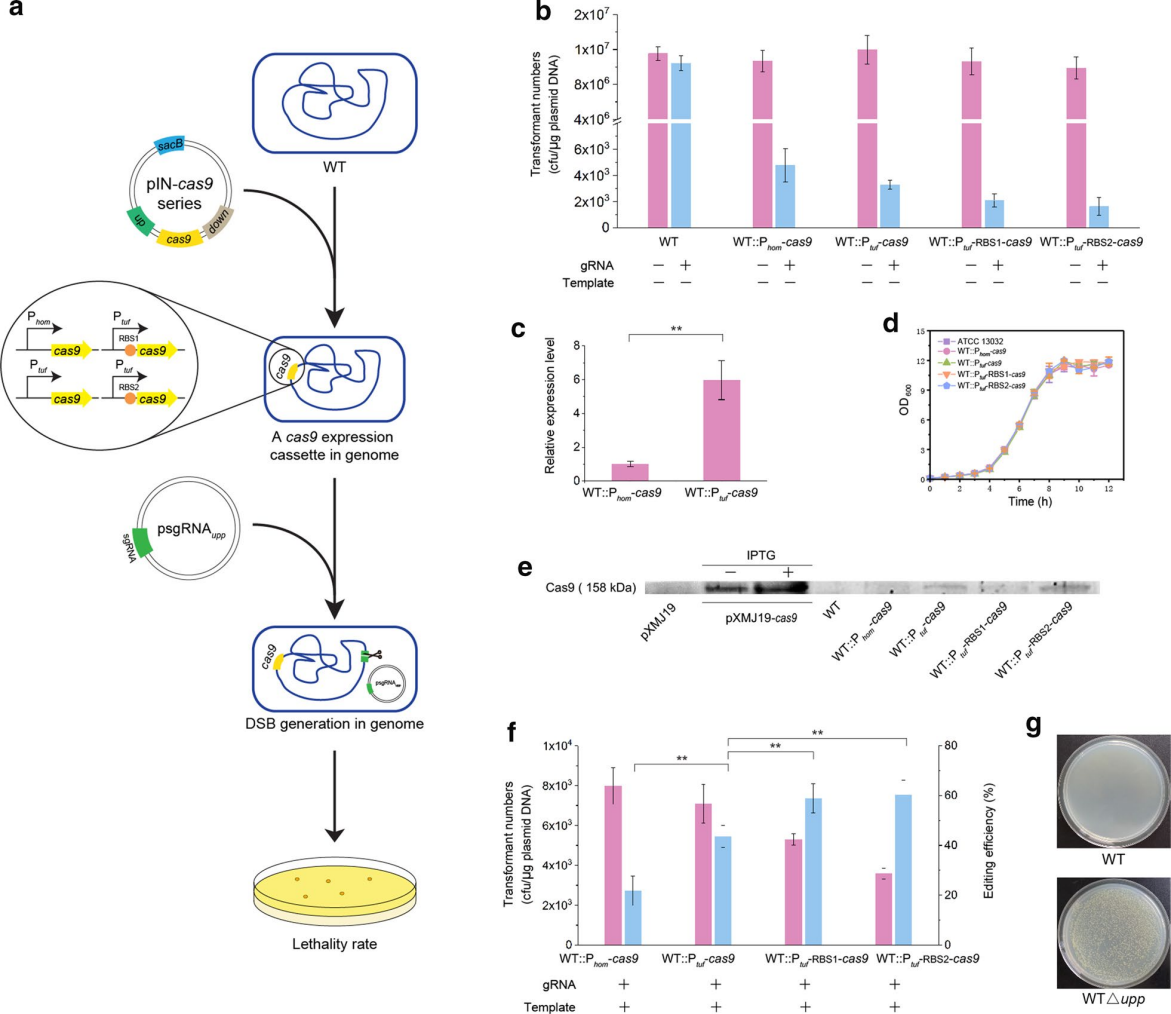
Optimization of CRISPR–Cas9 system by integrating *cas9* gene into the chromosome of *C. glutamicum*. **a** A chromosome-borne Cas9 expression cassette for lethality-based selection via sgRNA. The fragments of *Cas9* gene under the control of different promotors and RBSs were integrated into the genome using a series of pIN-*cas9* plasmids by two rounds of homologous recombination, respectively. psgRNA_*upp*_ harboring the sgRNA expression cassette was transformed into the Cas9-expressing strains by electroporation to detect lethality rate. **b** The numbers of transformants generated by electrotransformation of psgRNA_*upp*_ into WT, WT::P_*hom*_-*cas9*, WT::P_*tuf*_-*cas9*, WT::P_*tuf*_-rbs1-*cas9* and WT::P_*tuf*_-rbs2-*cas9* strains. pXMJ19ts was used as a control. **c** The relative transcription level of *cas9* gene. The transcription level of *cas9* in WT::P_*tuf*_-*cas9* was compared against that of *cas9* in WT::P_*hom*_-*cas9*. **d** The growth curves of *C. glutamicum* strains harboring different *cas9* expression cassettes. **e** The expression level of Cas9 protein detected by Western blotting. The expression of plasmid-borne Cas9 with ITPG induction was used as a positive control. **f** The numbers of transformants (red column) and editing efficiencies (blue column) generated by electrotransformation of pHAsgRNA_*upp*_ into *C. glutamicum* strains harboring different *cas9* expression cassettes. Significant differences in the data were determined using Student’s *t*-test (*P < 0.05, **P < 0.01). The data are derived from experiments performed at least three times, and the error bars represent the standard deviations. **g** Verification of inactivation of uracil phosphoribosyltransferase. The *upp*-deficient mutant was capable of growing on CGX agar plates containing 5-FU (20 μM)

To decrease the effect of Cas9 expression on cell viability, *cas9* gene under the control of a weak promoter P_*hom*_ and *rrnB* terminator was introduced into the wild-type *C. glutamicum* ATCC 13032 genome at the transposase (*Cgl1066*) locus, giving rise to strain WT::P_*hom*_-*cas9* (Fig. 1a and Additional file 1: Figure S1). To test the lethality of Cas9-induced DSB, the sgRNA targeting on *upp* gene (encoding uracil phosphoribosyltransferase) was designed and inserted into pXMJ19ts under the control of strong promoter P_*glyA*_, resulting in plasmid psgRNA_*upp*_. Transformation with psgRNA_*upp*_ yielded ~ 10^3^ cfu/μg plasmid DNA (Fig. 1b), indicating a high escape rate that is inadequate for eliminating wild-type cells. We assumed that the amount of Cas9 protein might be not enough to cleave target DNA effectively. Therefore, a strong promoter P_*tuf*_ was used to increase the transcription of chromosomal *cas9* gene. The mRNA level of *cas9* with P_*tuf*_ control increased by 4.93-fold compared to that under the control of P_*hom*_ (Fig. 1c), leading to a decrease in escape rate (Fig. 1b). Then, two ribosome binding sequences (RBS1 and RBS2) with increased translational initiation efficiencies were inserted into the front of the start codon of *cas9* gene, resulting in WT::P_*tuf*_-rbs1-*cas9* and WT::P_*tuf*_-rbs2-*cas9* strains (Fig. 1a). This led to the great decreases in the numbers of transformants (Fig. 1b), demonstrating the feasibility of CRISPR/Cas9-based counter-selection. The plasmid-borne Cas9 expression hindered cell growth of *C. glutamicum* in the previous report [27], therefore, we investigated the effect of chromosome-borne Cas9 expression on cell viability. When these strains harboring different expression cassettes of *cas9* gene were cultivated in BHIS medium, no difference in growth rate was observed (Fig. 1d). Furthermore, the expression level of chromosome-borne Cas9 was visibly lower than those of plasmid-borne IPTG-inducible Cas9 by Western blotting analysis, suggesting that the decreased expression of chromosome-borne Cas9 has little impact on cell growth (Fig. 1e).

To assess the editing efficiencies using sgRNA and different Cas9 expression cassettes for gene deletion in *C. glutamicum*, the *upp* gene encoding uracil phosphoribosyltransferase was chosen as the target gene. Since its inactivation led to the failure of conversion from 5-fluorouracile (5-FU) to a toxic product for cell growth and generated a 5-FU-resistant phenotype for convenient screening [8]. To delete *upp* gene, two homologous arms as repair templates were inserted into psgRNA_*upp*_ to construct pHAsgRNA_*upp*_. When transformed with pHAsgRNA_*upp*_ into WT::P_*hom*_-*cas9*, 21.79 ± 5.88% of the colonies (7/26, 4/26, 6/26) were confirmed by colony PCR to correctly edit *upp* gene (Fig. 1f and Additional file 1: Figure S2) and correspondingly gained the 5-FU resistance (Fig. 1g). For the next round genome editing, nearly all of colonies after an overnight incubation had lost the plasmid pHAsgRNA_*upp*_, indicating the convenience of a single-plasmid-based CRISPR–Cas9 system for genome editing in *C. glutamicum*. When transformed with pHAsgRNA_*upp*_ into WT::P_*tuf*_-*cas9*, WT::P_*tuf*_-rbs1-*cas9* and WT::P_*tuf*_-rbs2-*cas9*, the numbers of transformants decreased (Fig. 1f); however, the editing efficiency improved from 43.59 ± 4.44 to 60.26 ± 5.88% (Fig. 1f and Additional file 1: Figures S3–S5), indicating that the lethal efficiency of CRISPR/Cas9-based counter selection has a significant effect on editing efficiency for gene deletion.

### Introduction of RecET increases the editing efficiency of CRISPR–Cas9

**Fig. 2:**
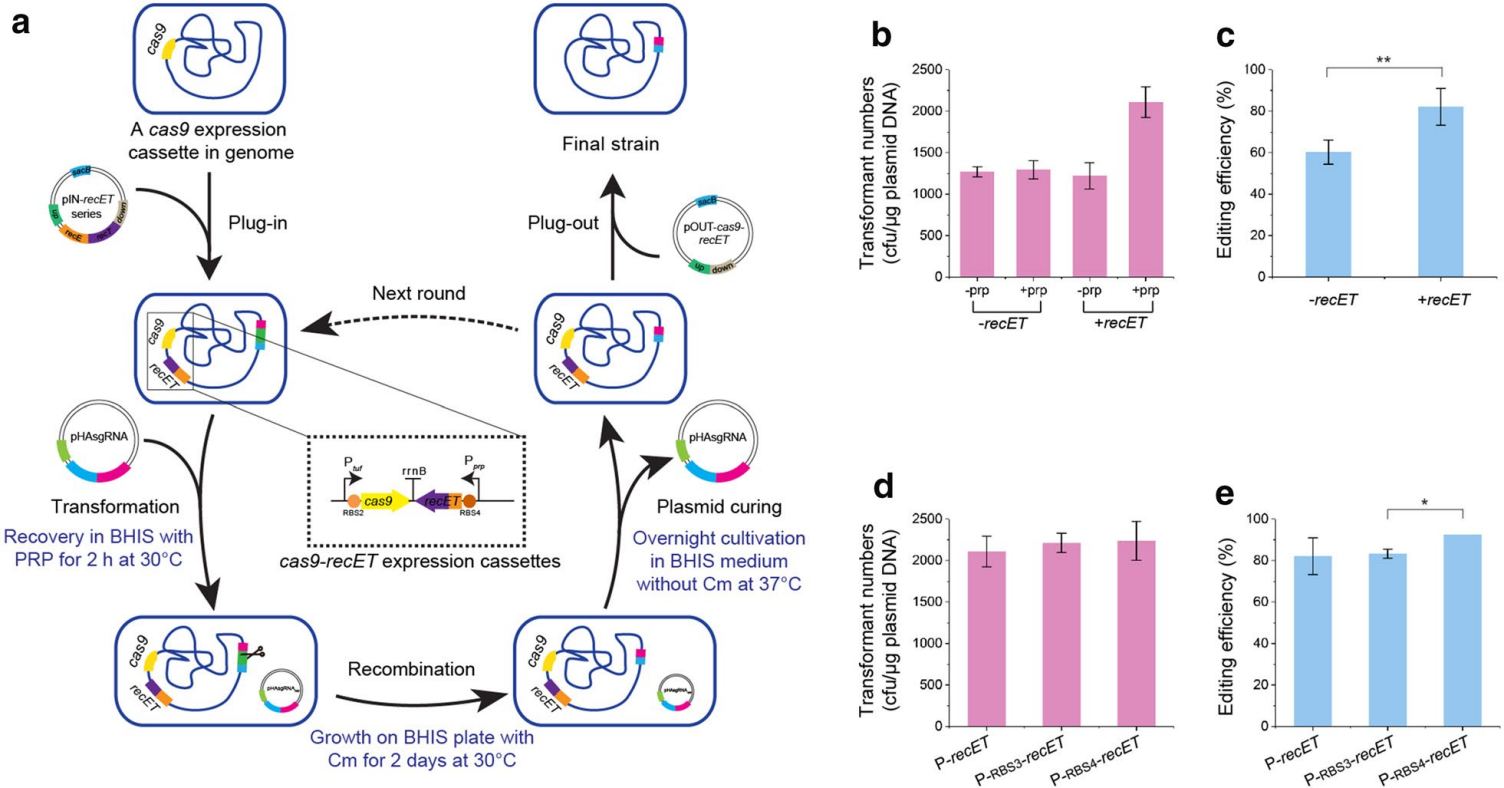
RecET-assisted CRISPR–Cas9 genome editing in *C. glutamicum*. **a** A flow-chart for a single-plasmid-based and RecET-assisted CRISPR–Cas9 editing method in *C. glutamicum*. *RecET* gene under control of a propionate-inducible promoter P_*prp*_ was integrated into WT::P_*tuf*_-rbs2-*cas9* via a series of pIN-*recET* plasmid by two rounds of homologous recombination. pHAsgRNA was transformed by electroporation into the strain harboring *cas9*–*recET* expression cassettes. After the recovery in BHIS supplemented with 0.5 g/L sodium propionate (PRP) at 30 °C for 2 h, cells were spread on chloramphenicol-resistant BHIS plate for 2-day cultivation. The positive colonies screened by colony PCR were subcultured in chloramphenicol-free BHIS medium at 37 °C overnight for plasmid curing. The edited strain without pHAsgRNA plasmids could be used for the next round of genome editing. Finally, the pOUT-*cas9*–*recET* carrying two homologous arms was transformed into the edited strain to plug out the chromosomal *cas9*–*recET* expression cassettes by two rounds of homologous recombination to get a final strain. **b** The number of transformants generated by electrotransformation of pHAsgRNA into WT::P_*tuf*_-rbs2-*cas9*::P_*prp*_-*recET* with or without propionate introduction. **c** Editing efficiencies under the noninducible or inducible RecET expression conditions. **d** The number of transformants generated by electrotransformation of pHAsgRNA into WT::P_*tuf*_-rbs2-*cas9*::P_*prp*_-rbs3-*recET* and WT::P_*tuf*_-rbs2-*cas9*::P_*prp*_-rbs4-*recET* strains. **e** Editing efficiencies in WT::P_*tuf*_-rbs2-*cas9*::P_*prp*_-rbs3-*recET* and WT::P_*tuf*_-rbs2-*cas9*::P_*prp*_-rbs4-*recET* strains. Significant differences in the data were determined using Student’s *t*-test (*P < 0.05, **P < 0.01). The data are derived from experiments performed at least three times, and the error bars represent the standard deviations

The edited mutants would be generated from double-crossover HR; therefore, the HR ability of *C. glutamicum* became a key factor in the editing efficiency of CRISPR/Cas9 system. To increase the probability of HR occurrence, *recET* from the defective Rac prophage under the control of inducible P_*prp*_ were introduced into the chromosome at the downstream of *cas9* locus, giving rise to the WT::P_*tuf*_-rbs2-*cas9*::P_*prp*_-*recET* (Fig. 2a and Additional file 1: Figure S6). As the inducible expression of RecET in the presence of propionate, the transformation with pHAsgRNA_*upp*_ produced 2.1 × 10^3^ cfu/μg plasmid DNA, which was a 70% increase compared to WT::P_*tuf*_-*cas9* (Fig. 2b). About 82.05 ± 8.88% of selected colonies were identified as the expected deletion of *upp* by colony PCR (Fig. 2c and Additional file 1: Figure S7). Two RBSs (RBS3 and RBS4) were inserted in the front of the start codon of *recET* to improve its translation initiation rate. The numbers of obtained colonies showed no significantly difference (Fig. 2d). As expected, editing efficiencies improved to 83.33 ± 2.22% in WT::P_*tuf*_-rbs2-*cas9*::P_*prp*_-rbs3-*recET* and 92.31 ± 0.00% in WT::P_*tuf*_-rbs2-*cas9*::P_*prp*_-rbs4-*recET* (Fig. 2e and Additional file 1: Figures S8, S9), respectively. These results suggested that introduction of *recET* gene increased the HR efficiency of *C. glutamicum*, resulting in a high editing efficiency. For convenience, WT::P_*tuf*_-rbs2-*cas9*::P_*prp*_-rbs4-*recET* strain was referred as EDT (Editable Type) in the following text. A detailed experimental protocol of the RecET-assisted CRISPR–Cas9 method had been shown in Additional file 2.

### Applicability of CRISPR–Cas9 editing method in *C. pekinense*

**Fig. 3:**
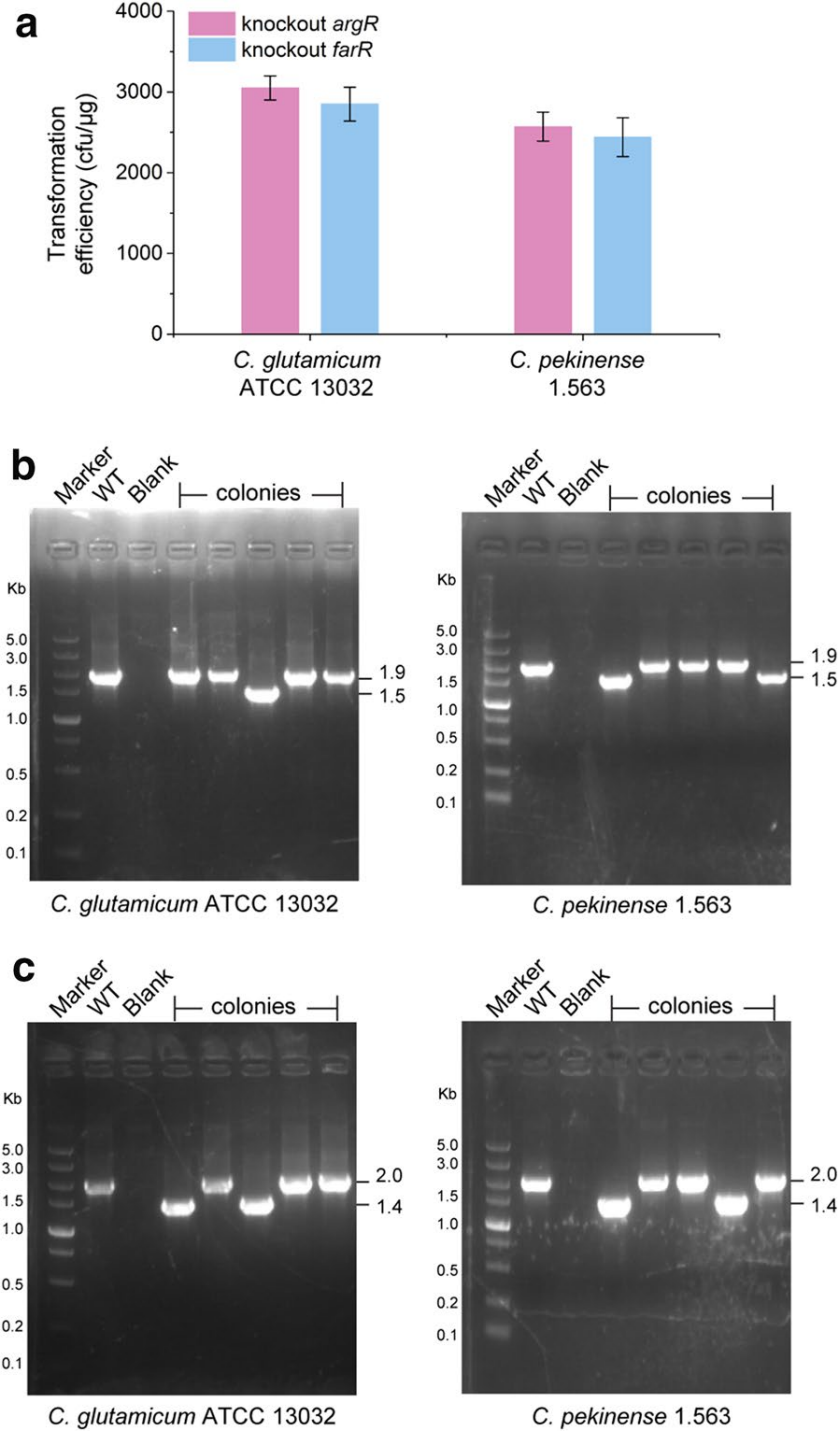
Applicability of CRISPR–Cas9 editing method in *C. pekinense*. **a** The transformation efficiency of pHAsgRNA_*argR*_ and pHAsgRNA_*farR*_ to *C. glutamicum* ATCC 13032 and *C. pekinense* 1.563 for knockout of *argR* and *farR* genes. **b** Colony PCR identification for *argR* deletion in *C. glutamicum* ATCC 13032 and *C. pekinense* 1.563. **c** Colony PCR identification for *farR* deletion in *C. glutamicum* ATCC 13032 and *C. pekinense* 1.563

To determine whether CRISPR–Cas9 system is applicable for other *C. glutamicum*-related bacteria, Cas9 expression cassette instead of RecET–Cas9 expression cassette was used to integrate into the chromosome due to the low frequency of integrating the 8.5-kb expression cassette of RecET–Cas9 using two rounds of homologous recombination in the chromosome. *C. pekinese* 1.563, a lysine producer screened by random mutagenesis was chosen as a candidate strain for genome editing [29]. Two sgRNA targeting *argR* encoding a regulator and *farR* encoding a global repressor were designed and used to construct pHAsgRNA_*argR*_ and pHAsgRNA_*farR*_ for deleting these two genes. Transformation with pHAsgRNA_*argR*_ produced 2.6 × 10^3^ cfu/μg plasmid DNA (Fig. 3a). The editing efficiency for *argR* deletion in *C. pekinense* 1.563 was 30.3%, which was similar to that in *C. glutamicum* ATCC 13032 (Fig. 3b and Additional file 1: Figure S10). And the deletion of *farR* was accomplished with a transformation efficiency of 2.4 × 10^3^ cfu/μg plasmid DNA and an editing efficiency of 30% in *C. pekinense* 1.563, respectively (Fig. 3a, c and Additional file 1: Figure S11). Therefore, our results indicated that the CRISPR–Cas9 system could be available for genome editing in other *Corynebacterium* species.

### Gene deletion and integration using RecET-assisted CRISPR–Cas9 method

**Fig. 4:**
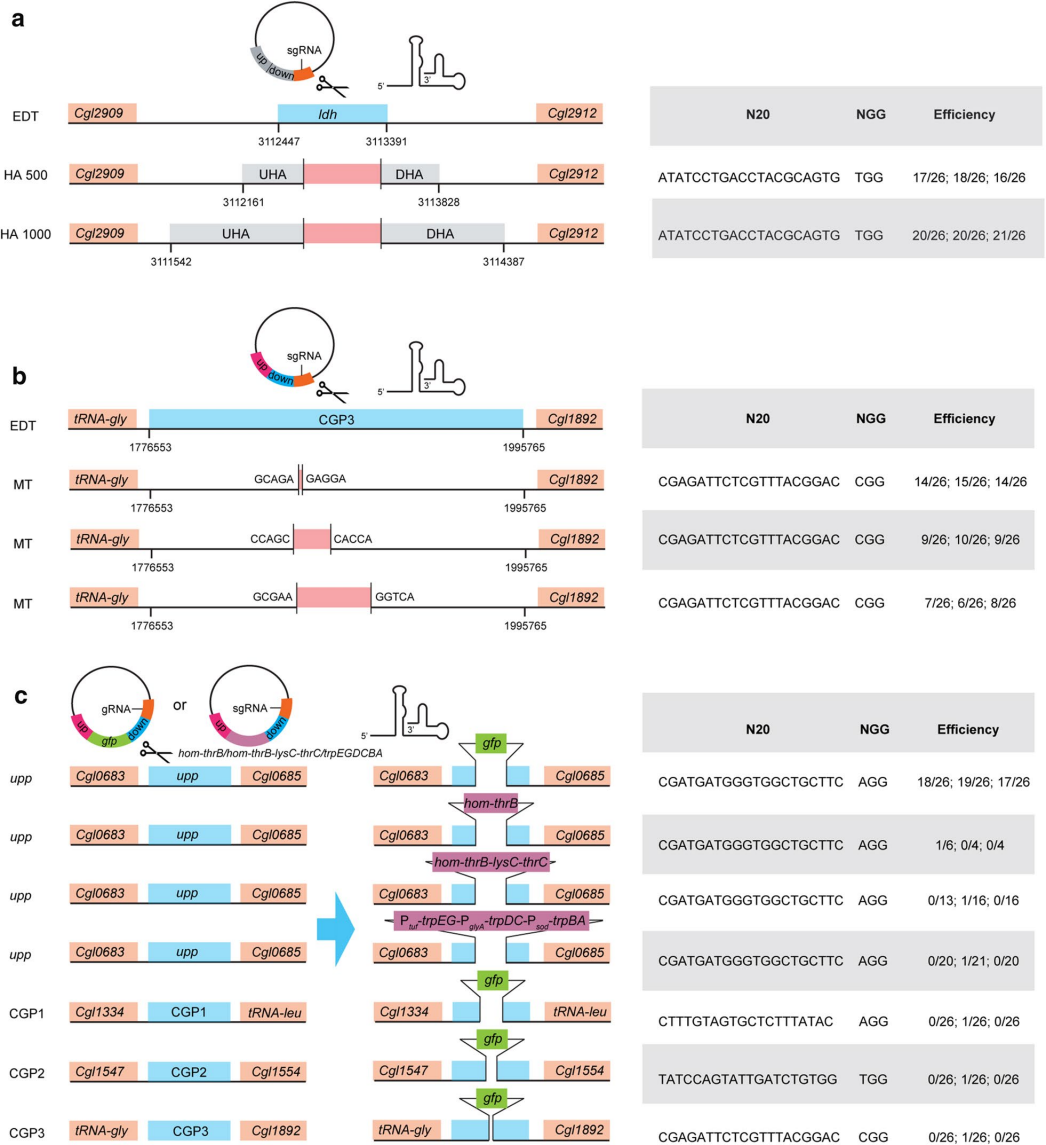
Gene deletion and insertion using the RecET-assisted CRISPR–Cas9 method. **a** Deletion of *ldh* gene using pHA500sgRNA_*ldh*_ and pHA 1000sgRNA_*ldh*_. **b** Deletion of 1-, 10- and 20-kb regions in CGP3 locus using pHAsgRNA_ΔCGP3–1kb_, pHAsgRNA_ΔCGP3–10kb_ and pHAsgRNA_ΔCGP3–20kb_. **c** Gene insertion in *upp*, CGP1, CGP2, CGP3 loci. For *upp* locus, both *gfp*, P_*tuf*_-*hom*-*thrB* and P_*tuf*_-*hom*-*thrB*-P_*glyA*_-*lysC*-*thrC* expression cassettes were chosen as the insertion fragments. For CGP1, CGP2 or CGP3 loci, *gfp* was chosen as the insertion gene

The method was further tested for efficiencies of gene deletion at different loci. Firstly, the inactivation of lactate dehydrogenase was performed by deleting *ldh* gene. Using the same sgRNA as that in the two-plasmid-based CRISPR/Cas9 method [27] for deleting *ldh* gene and the donor templates with 500 and 1000 bp HAs, pHA500sgRNA_*ldh*_ and pHA1000sgRNA_*ldh*_ were constructed and transformed into EDT, respectively (Fig. 4a). The editing efficiency with 1000 bp HAs (78.21 ± 2.22%) was higher than that with 500 bp HAs (65.38 ± 3.85%) (Additional file 1: Figures S12, S13), which was consistent with the effects of repair arm sizes on gene editing efficiency in the previous report [28]. To validate its efficiencies for deleting the fragments with variant sizes, CGP3, a 220 kb prophage [30], was chosen as the target to delete 1-, 10-, 20- and 200-kb regions using the same sgRNA_Δ__CGP3_ and different upstream and downstream HAs in EDT (Fig. 4b). The transformation with pHAsgRNA_Δ__CGP3–1kb_ into EDT produced 1.29 × 10^3^ cfu/μg plasmid DNA. Forty-three positive colonies were screened with a deletion efficiency of 55.13 ± 2.22% (Fig. 4b and Additional file 1: Figure S14). The transformation efficiencies with pHAsgRNA_Δ__CGP3–10kb_ and pHAsgRNA_Δ__CGP3–20kb_ decreased by 43% and 70%; and the deletion efficiencies of 10- and 20-kb regions were 35.90 ± 2.22 and 26.92 ± 3.85%, respectively (Additional file 1: Figures S15, S16). Unfortunately, the transformation with pHAsgRNA_Δ__CGP3–200kb_ only yielded ~ 10 colonies and the 200-kb deficient mutant was not obtained by colony PCR screening.

For gene insertion, 717-bp *gfpmut3a* gene as a candidate was inserted in *upp*, CGP1, CGP2 and CGP3 loci, respectively. The insertion of *gfp* at *upp* locus reached an efficiency of 69.23 ± 3.85% (Fig. 4c and Additional file 1: Figure S17). In contrast, the insertion of *gfp* at CGP1, CGP2 and CGP3 loci only showed editing efficiencies below 10.0% (Fig. 4c and Additional file 1: Figures S18–S20). Due to the effects of gRNA performance on the cutting effectiveness of the Cas9-sgRNA complex [31–33], we designed nine sgRNAs to target the different PAM sites locating in CGP1. However, the numbers of colonies harboring psgRNA_Δ__CGP1_ series vectors were significantly higher than those of colonies harboring the psgRNA_*upp*_ vector (data not shown), indicating that sgRNA performance might be a key factor to affect the editing efficiency of CRISPR–Cas9 system. Using the sgRNA targeting *upp* locus, a 2.5-kb P_*tuf*_-*hom*-*thrB* and 5.7-kb P_*tuf*_-*hom*-*thrB*-P_*glyA*_-*lysC*-*thrC* expression cassettes were inserted with the editing efficiencies of 7.14 and 2.22%, respectively (Fig. 4c and Additional file 1: Figure S21). Furthermore, only a positive mutant with a 7.5-kb P_*tuf*_-*trpEG*-P_*glyA*_-*trpDC*-P_*sod*_-*trpBA* expression cassette insertion at the *upp* locus was obtained through screening 61 transformants by colony PCR (Fig. 4c and Additional file 1: Figure S21). Using two different sgRNAs targeting the same locus between *cgl0900* and *cgl0901*, the 3.6-kb *lacZ* expression cassette was inserted into the genome with the efficiency of 16.67 ± 2.22 and 20.51 ± 2.22% (Additional file 1: Figure S22), respectively. Taken together, the differences in genomic locus and the length of inserted fragment had an obvious effect on the insertion efficiency.

### Site-directed mutation mediated by RecET-assisted CRISPR–Cas9 method

**Fig. 5:**
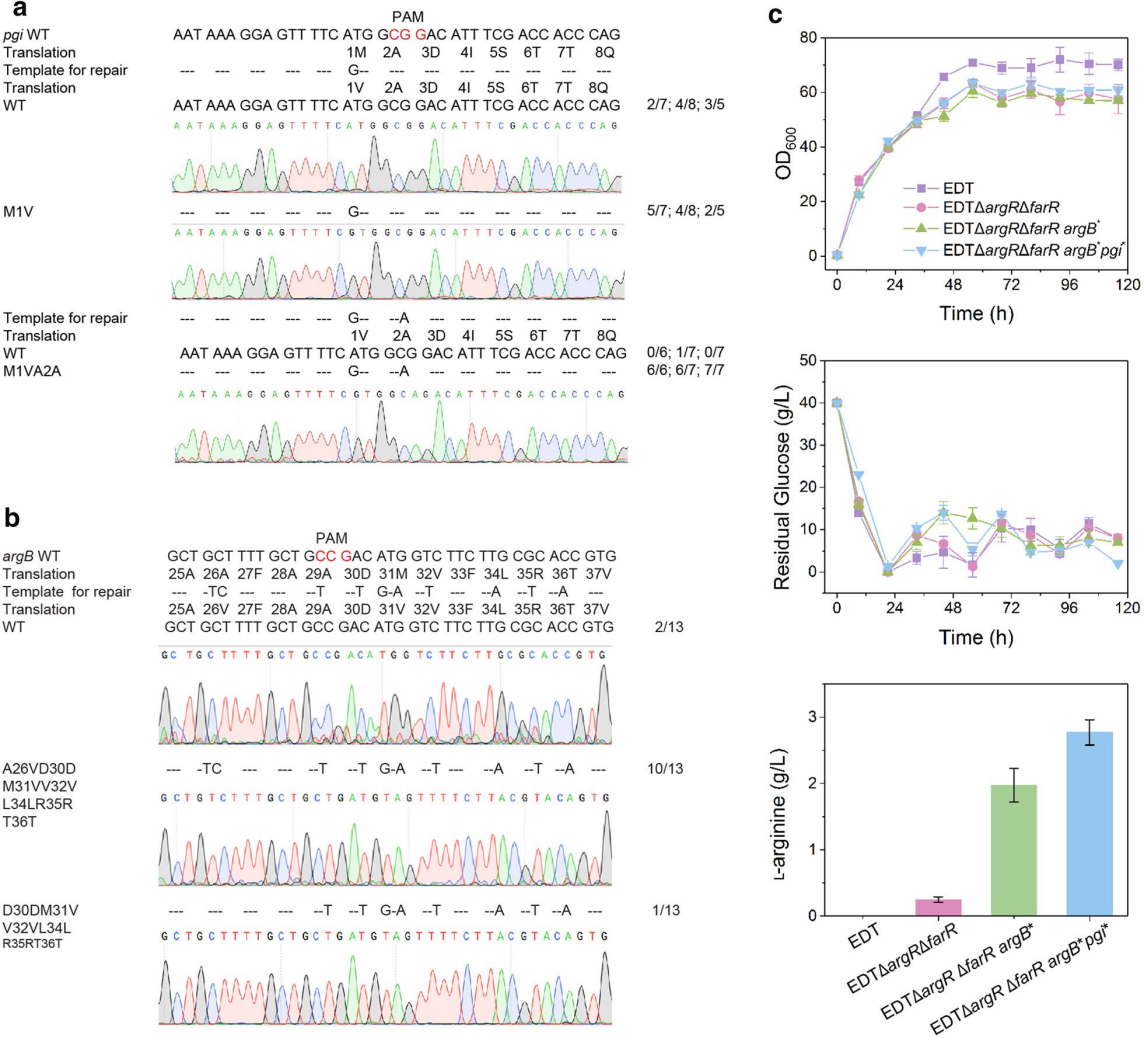
Point mutation using the RecET-assisted CRISPR–Cas9 method. **a** Verification of start codon replacement of *pgi* gene by sequencing. Plasmids pHAsgRNA_*pgi*1_ harboring the mutated start codon (ATG to GTG) in HAs and pHAsgRNA_*pgi*2_ harboring both the mutated start codon in HAs and the mutated nucleotides in PAM site (NGG to NAG) were transformed into EDT by electroporation. Cells were spread on chloramphenicol-resistant BHIS plate for cultivation. PCR amplifications were performed using random-selective colonies as the templates. (n/N): *n* number of correctly edited transformants, *N* number of tested transformants. **b** Mutants verification by sequencing *argB* gene. To replace the A26V and M31V at the same time, the repair templates were designed to contain 10 mutated base pairs, among which two nucleotides were mutated for amino acid substitution and the other eight nucleotides were mutated to prevent Cas9 cleavage. **c** The performance of EDT and its mutants for l-arginine production in the shake-flask cultivation. The data are derived from experiments performed at least three times, and the error bars represent the standard deviations

To evaluate the efficiency of site-directed mutation, the start codon of *pgi* gene were mutated from ATG to GTG to downregulate its expression, which led to the increase in the flux distribution towards pentose phosphate pathway [2]. Eleven colonies were identified to be correct edited by PCR amplification and DNA sequencing with an efficiency of 55.0% (Fig. 5a). When the mutation from CGG to CAG at the PAM was introduced into the repair template, the efficiency for *pgi* mutation increased to 95%. Then, this method was applied to introduce double amino acid mutations of A26V and M31V into the chromosomal ArgB to release the feedback inhibition by l-arginine [2]. The sgRNA targeting the sequence from 90th to 110th bp of *argB* gene and HA containing 10 bp mismatches were designed (Fig. 5b). Thirteen colonies were randomly selected for colony PCR identification. Ten colonies contained the total mutated sites in the argB* with an editing efficiency of 77% (Fig. 5b). Interestingly, one colony showed eight point mutations without A26V mutation, indicating that the double-crossover event happened at the downstream sites of A26 to repair the DSB.

Due to the potential instability of plasmid-borne Cas9, we further performed consecutive multiplex gene modifications in EDT to detect the stability of chromosome-borne Cas9. The deletion of *argR* showed an editing efficiency of 83.33% in EDT. The efficiency for *farR* deletion in EDTΔ*argR* was 75.00%. Two amino acid mutations of ArgB* was also performed in EDTΔ*argR*Δ*farR* with an editing efficiency of 77.06%. Shake flask cultivation showed that EDTΔ*argR*ΔfarRargB* accumulated 1.98 ± 0.26 g/L l-arginine. As knockdown of *pgi* expression in EDTΔ*argR*ΔfarRargB*, the growth and glucose consumption rates of EDTΔ*argR*ΔfarRargB*pgi_GTG_ were comparable to those of other strains (Fig. 5c), and l-arginine production reached 2.77 ± 0.19 g/L (Fig. 5c). These results indicated the stability of chromosome-borne Cas9 for iterative genome editing in *C. glutamicum*.

### Application of RecET-assisted CRISPR–Cas9 method to modify multiple genes for biosynthetic production of 1,2-propanediol

**Fig. 6:**
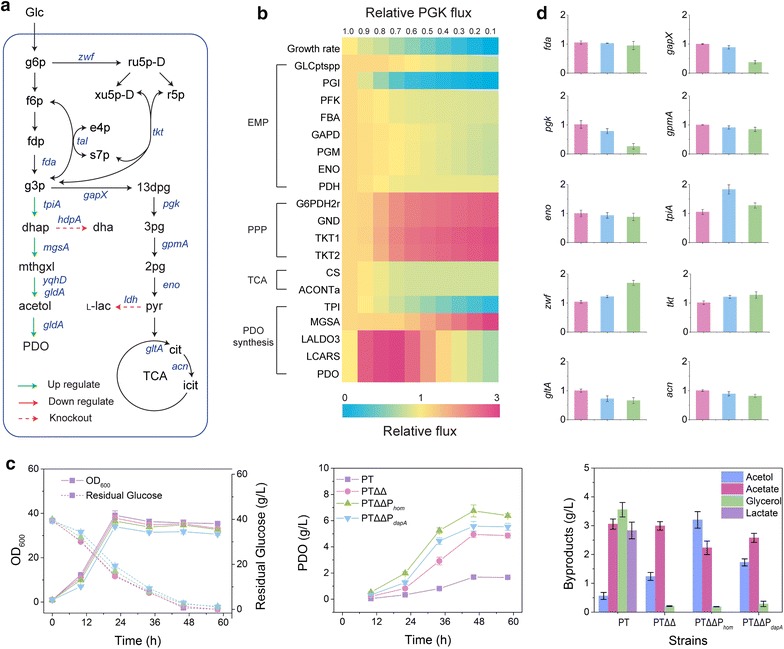
Multiple gene modifications for 1,2-propanediol production in *C. glutamicum* using the RecET-assisted CRISPR–Cas9 method. **a** Strategy of the multiple gene modifications for 1,2-propanediol production in *C. glutamicum*. **b** The impact of Pgk flux on cellular metabolism was investigated with decreasing relative Pgk fluxes (between 1.0 and 0.1). X-axis: relative Pgk flux (the ratio of the test Pgk flux to the native Pgk flux). Relative Pgk flux = 1.0 served as the control (or native GPD flux). Y-axis: relative flux of the pathways (the ratio of the test flux to the native flux). Yellow represents no change, red represents upregulation, and blue represents downregulation of the flux. **c** Flask cultivation of *C. glutamicum* strains for 1,2-propanediol (1,2-PDO) production. PTΔΔ: PT Δ*ldh*Δ*hdpA*; PTΔΔP_*hom*_: PTΔ*ldh* Δ*hdpA*P_*hom*_-*pgk*; PTΔΔP_*dapA*_: PTΔ*ldh*Δ*hdpA*P_*dapA*_-*pgk*. The data are derived from experiments performed at least three times, and the error bars represent the standard deviations. **d** Relative transcript levels of related genes in the different *C. glutamicum* strains at the exponential growth phase. Red column: PT Δ*ldh*Δ*hdpA*; blue column: PTΔ*ldh* Δ*hdpA*P_*hom*_-*pgk*; green column: PTΔ*ldh*Δ*hdpA*P_*dapA*_-*pgk*

*Corynebacterium glutamicum* have been engineered for 1,2-propanediol production by expressing the heterologous genes *mgsA* encoding methylglyoxal synthase, *gldA* encoding glycerol dehydrogenase and *yqhD* encoding alcohol dehydrogenase from *E. coli* [34, 35]. Genetic modification strategy for increasing 1,2-propanediol production have been predicted by *i*CW773 in our previous study [36]. The deletion of *hdpA* encoding dihydroxyacetone phosphate phosphatase and *ldh* encoding lactate dehydrogenase could decrease the formation of byproducts, glycerol and lactate, to increase the 1,2-propanediol production (Fig. 6a). Downregulation of *gap*, *pgk* and *gpmA* could decrease the split-flow of glyceraldehyde phosphate and drive more carbon fluxes towards 1,2-propanediol synthesis. Among three genes, *pgk* encoding phosphoglycerate kinase is essential for growth of *C. glutamicum* on carbon sources requiring glycolysis and gluconeogenesis, and a major control point in the glycolytic pathway during growth on glucose [37]. The effects of decreased Pgk flux on cell metabolism and 1,2-propanediol synthesis were simulated by *i*CW773 using FBA. The fluxes toward MgsA was significantly enhanced with the decrease in the relative Pgk flux, and the maximum extracellular 1,2-propanediol production was achieved when the relative Pgk flux was decreased by 30% compared with WT (Fig. 6b). Therefore, these genes were modified by RecET-assisted CRISPR–Cas9-mediated method to regulate the metabolic network for 1,2-propanediol synthesis (Fig. 6a).

Firstly, expression of *mgsA*, *gldA,* and *yqhD* genes in an artificial operon under the P_*tac*_ control from the plasmid pXMJ19-*mgsA*-*gldA*-*yqdD* gave rise to 1.69 ± 0.09 g/L 1, 2-propanediol accumulation with a yield of 0.10 mol/mol in a shake flask cultivation of PT (Fig. 6c). The efficiencies of *hdpA* and *ldh* deletion were 66.67 and 50.00%, respectively (Additional file 1: Figures S23, S24). Deletion of *hdpA* gene resulted in a 94.08% decrease in the extracellular glycerol accumulation in PTΔ*hdpA*Δ*ldh* (Fig. 6c). The lactate was almost undetectable in PTΔ*hdpA*Δ*ldh* that accumulated 4.95 ± 0.24 g/L 1,2-propanediol with a 2.0-fold increase in the product yield (0.30 mol/mol). The byproduct acetate was accumulated (2.99 ± 0.15 g/L), which was consistent with the previous report [34, 35]. To control the competing pathway for glyceraldehyde phosphate, two weak promoters, P_*hom*_ and P_*dapA*_, were used to construct pHAsgRNA_P*hom*_ and pHAsgRNA_P*dapA*_ for the promoter replacements to decrease the transcriptional level of *pgk* gene. The efficiencies of P_*hom*_ and P_*dapA*_ replacement were 66.67 and 83.33%, resulting in 22.66 and 74.41% decreases in the mRNA level of *pgk*, respectively (Fig. 6d). Meanwhile, the mRNA level of *gapX* decreased and the mRNA level of *tpiA* increased, which contributed to drive the flux toward the 1,2-propanediol synthetic pathway. However, no significant changes were observed in the mRNA levels of *fda*, *gpmA, gltA* and *acn*. When the mRNA level of *pgk* decreased, there were no significant differences in the growth and glucose consumption (Fig. 6d). PTΔ*hdpA*Δ*ldh*P_*hom*-_*pgk* and PTΔ*hdpA*Δ*ldh*P_dapA-_*pgk* produced 6.75 ± 0.46 and 5.57 ± 0.36 g/L 1,2-propanediol, respectively, which were 36.36 and 12.53% increase compared to PTΔ*hdpA*Δ*ldh*. Meanwhile, the precursor acetol showed the increasing trend similar to 1,2-propanediol and the byproduct acetate decreased by 25.42 and 13.71%, respectively (Fig. 6c). Finally, the downregulation of *pgk* improved the product yield to 0.41 mol/mol glucose, which was higher than the deletion of *hdpA* and *ldh* genes in the previous engineered *C. glutamicum* [34, 35]. For industrial applications, the *cas9*–*recET* expression cassettes were deleted from the chromosome of EDTΔ*ldh*Δ*hdpA*P_*hom*_-*pgk* using pOUT-*cas9*–*recET* plasmid to obtain a final engineered strain for 1,2-propanediol production (Additional file 1: Figure S25).

## Discussion

CRIPSR–Cas9 systems have been employed to be efficient biotechnology tools for genome editing in many bacteria [18–24]. Two-plasmid-based CRIPSR–Cas9 editing methods available for *C. glutamicum* require a high transformation efficiency to guarantee the co-occurrence of two plasmids transformation, resulting in their editing efficiencies comparable to those of other methods relying on two rounds of homologous recombination [26–28]. In this study, we developed a RecET-assisted CRISPR–Cas9-genome editing method for *C. glutamicum*. This approach employed a single plasmid to transform EDT, which enables transformation and plasmid curing procedures to be more simple and convenient than two-plasmid-based CRISPR–Cas9 method [27, 28]. Introduction of RecET increased the editing efficiency of this method, which is applicable for comprehensive genome editing, such as deletion of large DNA fragments, integration of multiplex gene expression cassettes, and promoter replacements as well as site-directed mutations.

In two-plasmid-based CRISPR–Cas9 methods, only the decreased expression level of SpCas9 under the control of inducible P_*tac*_ was successful to obtain transformants due to the potential toxicity of SpCas9 on cell viability [26–28]. Even so, the transformants harboring pCas9 grew at a lower rate than the wild-type strain [28]. Moreover, plasmid-borne Cas9 occurred spontaneous mutation, resulting in editing failure [27]. To decrease the copies of *cas9* gene, it was integrated into the chromosome under the control of *P*_tuf._, whose strength was lower than that of P_*tac*_ [38]. Western blotting analysis testified that the expression of chromosome-borne Cas9 maintained at the relative lower level compared to plasmid-borne Cas9, which might be the reason for no impacts of chromosome-borne Cas9 expression on cell growth. Furthermore, no off-target mutations could be detected in *C. glutamicum* expressing plasmid-borne Cas9 protein by genome sequencing [28], indicating that the chromosome-borne Cas9 might generate a very low probability of off target.

The induction of DSB on chromosome by functional Cas9-sgRNA complex is lethal to some bacteria lacking NHEJ pathway like eukaryotes [14, 39, 40]. For *C. glutamicum*, the co-transformation of CRISPR–Cas9 system with either dsDNA with different HA lengths or ssDNA as editing templates had been testified to be insufficient to repair the DSB in the absence of RecT [26]. The dsDNAs on the plasmids was unable to repair the DSB unless expressing the phage-derived recombinases (RecET) in *E. coli* [18], indicating that homologous recombination efficiency is a key factor that affects the editing efficiency of CRISPR–Cas9 system. However, plasmid-borne dsDNA could repair DSB in *C. glutamicum* expressing the chromosome-borne Cas9, suggesting that the introduction of DSB by CRISPR–Cas9 system increased the HR of the damaged DNA as reported elsewhere [18]. Introduction of exogenous RecET into *C. glutamicum* chromosome promoted the frequency of double-crossover event between chromosomal DSB region and repair templates [8, 25, 26, 41–43], which contributed to genome editing mediated by CRISPR–Cas9.

Compared to other CRISPR–Cas9 genome editing methods, our method has some advantages in transformation efficiency and multiple genetic manipulation, indicating a promising application in iterative genome editing of *C. glutamicum*. A high transformation efficiency is necessary for two-plasmid-based CRISPR–Cas9-coupled recombineering system to co-transform single-stranded oligodeoxyribonucleotides (ssODN) and Cas9-sgRNA vector into *C. glutamicum* [26]. A one-step electro-transformation strategy to co-transform pCas9 and pgRNA had been adopted to reduce false positives formation [27]. The low frequency of co-transformation led to less transformants. In contrast, transformation with a single-plasmid harboring sgRNA and HAs produced 10^3^ cfu/μg plasmid DNA. This transformation efficiency could be achieved easily and reproducibly for *C. glutamicum* at laboratory level, suggesting a universal application of a single-plasmid-based CRISPR–Cas9 system. Moreover, both pCas9 and pgRNA plasmids need to be removed in the absence of antibiotic selection pressure for several subcultures and then be co-transformed together for the next round of genome editing [27]. In contrast, a single-plasmid is easy to be cured by overnight cultivation and another new editing plasmid can be efficiently transformed for the next round of genome editing, indicating the convenience of our method for iterative genome editing. As for gene deletion, the efficiencies of *ldh* deletion using this method are slightly higher than that using two-plasmid-based CRISPR–Cas9 method (Additional file 1: Table S1) [27]. However, the deletion of 200-kb fragment was unsuccessful, which might be attributed to the decreased efficiency for gene deletion with the increase in gene length as reported previously [9]. Furthermore, CRISPR–Cas9 method relied on a double-crossover event, which simultaneously occurred between the DSB region and donor DNA template after the cleavage of Cas9-sgRNA complex. DSB was generated in the middle part of 200-kb fragment, while the strand exchange of HR occurred between the two sides of 200-kb fragment and plasmid-borne HAs. Therefore, a long spacing between DSB and HAs gave rise to a rare frequency of HR relying on a double-crossover event. However, TCCRAS method could achieve the deletion of 179.8-kb fragment in our previous report, due to a little high probability in the successive occurrence of a single-crossover event between the chromosomal homologous regions and plasmid-borne HAs under the screening pressure. Despite the numbers of *lacZ* insertion transformants (13 colonies) obtained by this method were higher than those (4 colonies) screened by two-plasmid-based CRISPR–Cas9 method, the insertion efficiency using this method was relatively low. It attributed to the differences in transformation efficiency and lethality of Cas9 between two methods. A large number of colonies were obtained as transformation with a single plasmid compared to the co-transformation with two plasmids. Meanwhile, the abundance of chromosomal-borne Cas9 maintained at a relatively lower level than that of plasmid-borne Cas9, resulting in the low lethality of chromosomal-borne Cas9 and the high numbers of false positive colonies. Furthermore, the integration efficiencies mediated by CRISPR–Cas9 at different chromosomal position in *E. coli* were variable due to the differences of cutting effectiveness of the Cas9-sgRNA complex [44]. It has also been demonstrated that different sgRNA can generate a great impact on editing efficiency in *C. glutamicum*. Therefore, the sgRNA less than 60% GC content is recommended to design for increasing the editing efficiency of CRISPR–Cas9 system in *C. glutamicum* [28].

## Conclusions

In this study, we developed a RecET-assisted CRISPR–Cas9 genome editing method for *C. glutamicum*. This method took advantage of the chromosome-borne Cas9 to reduce the instability of plasmid harboring Cas9 and utilized RecET to increase the frequency of homologous recombination. Transformation efficiency was improved by using a single-plasmid harboring a sgRNA and repair template, resulting in high efficiencies in gene deletion, insertion and site-specific mutation. In silico model-predicted target genes were modified using this method, and the final engineered strain produced 6.75 ± 0.46 g/L 1, 2-propanediol. This method will boost the metabolic engineering of *C. glutamicum* to produce the desired bio-based products from renewable biomass.

## Methods

### Bacterial strains, plasmids and cultivation conditions

*Corynebacterium glutamicum* ATCC 13032 (American Type and Culture Collection, Manassas, VA, USA) was used as the parental strain for genome editing. The *E. coli* strain EC135 [45] was used as the cloning host for plasmid construction. The pK18*mobsacB* was used for the introduction of *cas9* and *recET* genes into the chromosome. The pXMJ19ts with a temperature-sensitive replication origin [46] was applied to express sgRNA cassette and carry the DNA repair template for gene deletion, insertion and site-directed mutation. All of the strains and plasmids used in this study are listed in Additional file 1: Tables S2 and S3. *E. coli* strains were cultured in Luria–Bertani medium at 37 °C. *C. glutamicum* strains were cultured in brain heart infusion medium at 30 °C. When necessary, antibiotics were added at the following concentrations: 50 μg/mL kanamycin or 20 μg/mL chloramphenicol for *E. coli* and 25 μg/mL kanamycin, 10 μg/mL chloramphenicol for *C. glutamicum*.

### DNA manipulations

DNA manipulations were performed using the standard cloning techniques [47], and all primers for plasmids construction are shown in Table S4. Polymerase chain reaction (PCR) amplifications were performed with Q5 High-Fidelity DNA Polymerase (NEB) and Taq DNA Polymerase (Tiangen) for cloning and colony PCR identification, respectively. Gibson Assembly were conducted with NEBuilder Master Mix (NEB). DNA gel Extraction Kit (Tiangen) was used to concentrate the DNA fragment and NanoDrop 2000 UV-Vis Spectrophotometer (Thermo Scientific) was used to determine the concentration of DNA fragment for Gibson Assembly [48].

### Insertion of Cas9 and RecET expression cassettes into chromosome

To introduce *cas9* gene into the chromosome, the *cas9* gene was amplified using pCas as the template with primers P5/P6. P_*hom*_, the upstream homologous arms (HA), and downstream HA were amplified using *C. glutamicum* ATCC 13032 genome as template with primers P3/P4, P1/P2 and P9/P10, respectively. The terminator *rrnB* was amplified from pXMJ19 with primers P7/P8. These fragments together with linearized pK18*mobsacB* were purified and recovered for Gibson assembly to construct pIN-P_*hom*_-*cas9*. To construct pIN-P_*tuf*_-*cas9*, pIN-P_*tuf*_-rbs1-*cas9* and pIN-P_*tuf*_-rbs2-*cas9*, the same procedures were applied using the indicated primers shown in Additional file 1: Table S4. Then, pIN-P_*hom*_-*cas9*, pIN-P_*tuf*_-*cas9*, pIN-P_*tuf*_-rbs1-*cas9* and pIN-P_*tuf*_-rbs2-*cas9* were transformed to the competent cells of *C. glutamicum* ATCC 13032 by electroporation, respectively. Screening for the first and second recombination events were performed as described previously [7]. Kanamycin-sensitive and sucrose-resistant clones were verified by colony PCR and DNA sequencing to obtain WT::P_*hom*_-*cas9*, WT::P_*tuf*_-*cas9*, WT::P_*tuf*_-rbs1-*cas9*, WT::P_*tuf*_-rbs2-*cas9* strains (Additional file 1: Figure S1).

To integrate *recET* gene into the chromosome, *E. coli* W3110 genome was used as a template to amplify *recET*(anti) with primers P33/P34. WT::P_*hom*_-Cas9 genome was used as a template to amplify *cas*_tail_*rrnB* with primers P31/P32. WT::P_*hom*_-Cas9 genome was used as a template to amplify P_*prp*_(anti) and down HA with primers P35/P36 and P37/P38. These fragments together with linearized pK18*mobsacB* were purified and recovered for Gibson assembly to construct pIN-P_*prp*_-*recET*. The same procedures were performed to construct pIN-P_*prp*_-rbs3-*recET* and pIN-P_*prp*_-rbs4-*recET* using the indicated primers in Additional file 1: Table S4. Then, pIN-P_*prp*_-*recET*, pIN-P_*prp*_-rbs3-*recET* and pIN-P_*prp*_-rbs4-*recET* were transformed to WT::P_*tuf*_-rbs2-*cas9* by electroporation. Screening for the first and second recombination events were performed as described previously [7]. Kanamycin-sensitive and sucrose-resistant clones were verified by colony PCR and DNA sequencing to obtain WT::P_*tuf*_-rbs2-*cas9*::P_*prp*_-*recET*, WT::P_*tuf*_-rbs2-*cas9*::P_*prp*_-rbs3-*recET* and WT::P_*tuf*_-rbs2-*cas9*::P_*prp*_-rbs4-*recET* strains (Additional file 1: Figure S6).

### Construction of sgRNA expression plasmids

To construct sgRNA expression plasmid, the 20 bp spacer sequence specific for each target was synthesized in primers and the sgRNA scaffold was amplified by PCR using pTargetF as the template. The P_*glyA*_ for sgRNA transcription and the upstream and downstream HAs for DSB repair were amplified using genome of *C. glutamicum* ATCC 13032 as the template. The pXMJ19 was used as a template to amplify mutated pXMJ19ts using primers P11/P12. The PCR product was digested with *Dpn*I and transformed into *E. coli* to obtain pXMJ19ts [49]. All the fragments and the linearized pXMJ19ts as the backbone were fused by Gibson Assembly [48], and followed by transformation in EC135 [45].

To construct pHAsgRNA_*upp*_, pHAsgRNA_*nc*_, pHAsgRNA_*argR*_, pHAsgRNA_*farR*_, pHAsgRNA_*argB*10_, pHAsgRNA_*argB*3_, p*hom*-*thrB*HAsgRNA_*upp*_, pHAsgRNA_*pgi*1_, pHAsgRNA_*pgi*2_, pHA500sgRNA_*ldh*_, pHA1000sgRNA_*ldh*_, pHAsgRNA_ΔCGP3–1kb_, pHAsgRNA_ΔCGP3–10kb_, pHAsgRNA_ΔCGP3–20kb_, p*gfp*HAsgRNA_*upp*_, p*gfp*HAsgRNA_CGP1_, p*gfp*HAsgRNA_CGP2_, p*gfp*HAsgRNA_CGP3_, p*lacZ*HAsgRNA_*cgl0900*–*cgl0901*-1_, p*lacZ*HAsgRNA_*cgl0900*–*cgl0901*-2_, pHAsgRNA_*hdpA*_, pHAsgRNA_ΔP*pgk::Phom*_, and pHAsgRNA_ΔP*pgk*::P*dapA*_, the above-mentioned procedures were conducted using the indicated primers in Additional file 1: Table S4. The integrity of all plasmids constructed in this study was confirmed by sequence analysis.

### Gene editing using the RecET-assisted CRISPR–Cas9 method

We established a single-plasmid-based approach for CRISPR-assisted genome editing in *C. glutamicum*. The competent cells of *C. glutamicum* harboring the chromosomal *cas9*–*recET* expression cassettes were prepared as previously described [50]. 100 ng of the pHAsgRNA were transformed by electroporation into the competent cells, and then cells were recovered in the BHIS medium supplementing with 0.5 g/L sodium propionate for RecET induction at 30 °C for 2 h. After that, cells were spread on BHIS plate containing 10 μg/mL chloramphenicol to incubate for 2 days. The transformants were screened by colony PCR with corresponding primers and verified by DNA sequencing. c.f.u. was normalized for per 1 μg DNA to calculate the transformation efficiency. The editing efficiency is defined as the ratio of correctly edited colonies to the total tested colonies [9], and calculated using the following formula:

$${\text{Editing efficiency}} = {\text{Number of correctly edited colonies/Number of tested colonies}} .$$

After screening the colonies with correct editing, cells were incubated in BHIS medium at 37 °C for overnight cultivation and plated to BHIS plates without chloramphenicol to cure the plasmid pHAsgRNA. To determine the curing efficiency, colonies were transferred to LB agar plates with and without chloramphenicol for screening the chloramphenicol sensitive colonies. Due to the high efficiency of plasmid curing, colonies could be used for the next round of RecET-assisted CRISPR–Cas9 genome editing without chloramphenicol sensitivity test.

### Plug out *cas9*–*recET* expression cassettes from chromosome

For plug out the chromosomal *cas9*–*recET* expression cassettes, the upstream and downstream homologous arms (HA) of chromosome-borne *cas9*–*recET* expression cassettes were amplified using P188/P189 and P190/P191, and then ligated into the pK18*mobsacB* by Gibson Assembly with NEBuilder Master Mix (NEB) to construct pOUT-*cas9*–*recET*. Subsequently, the pOUT-*cas9*–*recET* plasmid was transformed into the edited strains to delete *cas9*–*recET* expression cassettes by two rounds of homologous recombination to get a final strain.

### CRISPR/Cas9-assisted genome editing in *C. pekinense*

A lysine-producing *C. pekinense* 1.563 screened by random mutagenesis was used to verify the application of the CRISPR/Cas9 system. To insert *cas9* expression cassette, the non-replicated pIn-P_*tuf*_-RBS2-*cas9* plasmid was electroporated into the competent cells of *C. pekinense* 1.563 to obtain 1.563::P_*tuf*_-RBS2-*cas9* strain by two rounds of recombination events. To delete *argR* and *farR* genes, 100 ng of the pHAsgRNA_*argR*_ and pHAsgRNA_*farR*_ plasmids were then transformed by electroporation into 1.563::P_*tuf*_-RBS2-*cas9,* respectively. Cells were recovered in the resuscitation medium, spread on BHIS plates supplemented with 10 μg/mL chloramphenicol and then incubated for 2 days. The procedures for transformant screening and plasmid curing were performed as above described in *C. glutamicum* ATCC 13032.

### RNA preparation and quantitative RT-PCR

In order to determine the level of *cas9* of two *cas9*-expressing strain, i.e. WT::P_*hom*_-*cas9* and WT::P_*tuf*_-*cas9*, quantitative reverse transcription PCR (qRT-PCR) was performed using total RNA samples. Total RNA was isolated from the cells with the RNAprep Pure Cell/Bacteria Kit (Tiangen, China). The cDNA from approximately 300 ng of RNA was prepared using the specific primers listed in Additional file 1: Table S4 and the FastQuant RT Kit (Tiangen, China). The *rpoB* gene, which encodes RNA polymerase β subunit, was used as the reference gene [51]. The primers used for RT-PCR were listed in Additional file 1: Table S4. The qRT-PCR was performed with the GoTaq qPCR master mix (Promega, USA) in a 20-μL mixture using the LightCycler^®^ 96 Real-Time PCR System (Roche, Switzerland). The data analyses were performed using the LightCycler^®^ 96 software (Roche, Switzerland).

### Western blotting

*Corynebacterium glutamicum* cells were resuspended in 1 mL of phosphate-buffered saline (PBS) and disrupted using an Ultrasonic Cell Disruptor. The supernatants were collected by centrifugation at 12,000 rpm at 4 °C for 30 min. Protein samples were separated by Sodium dodecyl sulfate-polyacrylamide gel electrophoresis (SDS-PAGE) using a 10% running gel and 5% stacking gel and transferred to a nitrocellulose membrane (PVDF) by electroblotting. The proteins were probed with rabbit polyclonal antibody against Cas9. The blots were visualized with a peroxidase-coupled goat anti-rabbit secondary antibody and an ECL color development reagent (GE, USA).

### Modification of l-arginine synthetic network in *C. glutamicum*

The strain WT::P_*tuf*_-rbs2-*cas9*::P_*prp*_-rbs4-*recET* (EDT) was used to construct the engineered strains for l-arginine production. The plasmid pHAsgRNA_*argR*_ and pHAsgRNA_*farR*_ were iteratively transformed by electroporation to delete *argR* and *farR* genes encoding two transcriptional regulator as the above-described procedure, resulting in the strains EDTΔ*argR* and EDTΔ*argR*Δ*farR*. pHAsgRNA_*argB*10_ was transformed by electroporation into the strain EDTΔ*argR*Δ*farR* for site-directed mutation of chromosomal *argB* to release the feedback inhibition by l-arginine. The plasmid pHAsgRNA_*pgi*2_ was transformed by electroporation into the strain EDTΔ*argR*ΔfarRargB* to change the start codon ATG to GTG for *pgi* knockdown, resulting in the strain ETΔ*argR*ΔfarRargB*pgi_GTG_.

### Modification of 1,2-propanediol synthetic network in *C. glutamicum*

The strain WT::P_*tuf*_-rbs2-*cas9*::P_*prp*_-rbs4-*recET* (EDT) was used to construct the engineered strains for 1,2-propanediol  production. The plasmid pHAsgRNA_*ldh*_ and pHAsgRNA_*hdpA*_ were iteratively transformed by electroporation to delete *ldh* and *hdpA* as the above-described procedure, resulting in the strain EDTΔ*ldh*Δ*hdpA*. pHAsgRNA_Phom_ and pHAsgRNA_PdapA_ were transformed by electroporation into the strain EDTΔ*ldh*Δ*hdpA* separately for promoter replacement of *pgk* to increase the flux towards 1,2-propanediol, generating the strains EDTΔ*ldh*Δ*hdpA*P_*hom*_-*pgk* and EDTΔ*ldh*Δ*hdpA*P_*dapA*_-*pgk*. pXMJ19-*mgsA*-*gldA*-*yqhD* was transformed by electroporation into the strains EDT, EDTΔ*ldh*Δ*hdpA*, EDTΔ*ldh*Δ*hdpA* P_*hom*_-*pgk* and EDTΔ*ldh*Δ*hdpA*P_*dapA*_-*pgk*, giving rise to the strains PT, PTΔ*ldh*Δ*hdpA*, PTΔ*ldh*Δ*hdpA*P_*hom*_-*pgk* and PTΔ*ldh*Δ*hdpA*P_*dapA*_-*pgk*, respectively. To obtain a final engineered strain for 1,2-propanediol production, the plasmid pOUT-*cas9*–*recET* was transformed into the strain EDTΔ*ldh*Δ*hdpA*P_*hom*_-*pgk* by electroporation to plug out the *cas9*–*recET* expression cassette in the chromosome to get a final engineered 1,2-PDO strain.

### Shake flask cultivation

For l-arginine fermentation, *C. glutamicum* strains were precultured in the CGIII seed medium [52] at 30 °C and 220 rpm until the OD_600_ reached 10. One milliliter of seed culture was inoculated in 500-mL baffled shake flasks containing 30 mL CGXII medium supplemented with 40 g/L glucose and 2 g/L yeast extract. The cells were grown at 30 °C, 220 rpm and the pH was maintained at 7.0–7.2 by supplementation with ammonia.

For 1,2-propanediol fermentation, *C. glutamicum* strains were precultured in CGIII seed medium. The cells of an overnight preculture were inoculated into 40-mL fermentation cultures in 500-mL baffled flasks by transferring appropriate volume for a final optical density of 1.0. Fermentation medium contained (g/L): glucose 40, yeast extract 2, MgSO_4_·7H_2_O 0.25, (NH_4_)_2_SO_4_ 10, KH_2_PO_4_ 1, K_2_HPO_4_ 1, FeSO_4_·7H_2_O 0.01, MnSO_4_·H_2_O 0.01, ZnSO_4_·7H_2_O 0.001, CuSO_4_ 0.0002, NiCl_2_·6H_2_O 0.00002, biotin 0.0002, thiamine 0.0002 and 3-(*N*-morpholino) propanesulfonic acid (MOPS) 42. The cells were grown at 30 °C and 160 rpm. The expressions of *mgsA*, *gldA,* and *yqhD* genes were induced by adding 1 mM isopropyl-β-d-thiogalactopyranoside (IPTG) at 6 h. The pH was maintained at 7.0–7.2 by supplementation with ammonia.

### Analytical methods

The glucose and lactate concentrations were assayed using an SBA-40D biosensor analyzer (Institute of Biology of Shandong Province Academy of Sciences, Shandong, China). The cell concentration was determined by measuring the absorbance at 600 nm (OD_600_) using a spectrophotometer (V-1100D; Mapada Instruments, Shanghai, China). To quantify l-arginine, high-pressure liquid chromatography (HPLC) was performed using an Agilent 1260 series chromatography system equipped with a ZORBAX Eclipse AAA column (4.6 mm × 150 mm, 5 μm; Agilent) at 40 °C. Fluorescence detection was carried out after automatic pre-column derivatization with *O*-phthalaldehyde (OPA) using a variable wavelength detector (VWD) at 338 nm according to the instruction manual. The 1,2-propanediol, acetol, glycerol and acetate in the shake flask were analyzed using a Gas Chromatograph Mass Spectrometer (GCMS-QP2010 Ultra, Shimadzu, Japan) connected to an AOC-20i Auto-sample using a TG-WAXMS (length: 30 m; I.D.: 0.25 mm; film: 0.25 μm) (Thermo Scientific, USA). The samples were directly diluted 1:10 with methanol and centrifuged by 12,000×*g* for injection. The operating set up followed as the previous report [35]. The concentrations of 1,2-propanediol, acetol, glycerol and acetate were determined according to a calibration curve with an external standard. The peaks were identified by retention time and quantified using the intensity of one specific *m/z* value.

## Additional files

**Additional file 1: Figure S1.** Colony PCR verification of *cas9* integration. **Figure S2.** Colony PCR verification of *upp* deletion in WT::P_*hom*_-*cas9*. **Figure S3.** Colony PCR verification of *upp* deletion in WT::P_*tuf*_-*cas9*. **Figure S4.** Colony PCR verification of *upp* deletion in WT::P_*tuf*_-rbs1-*cas9*. **Figure S5.** Colony PCR verification of *upp* deletion in WT::P_*tuf*_-rbs2-*cas9*. **Figure S6.** Colony PCR verification of *recET* integration. **Figure S7.** Colony PCR verification of *upp* deletion in WT::P_*tuf*_-rbs2-*cas9*::P_*prp*_-*recET*. **Figure S8.** Colony PCR verification of *upp* deletion in WT::P_*tuf*_-rbs2-*cas9*::P_*prp*_-rbs3-*recET*. **Figure S9.** Colony PCR verification of *upp* deletion in WT::P_*tuf*_-rbs2-*cas9*::P_*prp*_-rbs4-*recET* (EDT). **Figure S10.** Colony PCR verification of *argR* deletion in EDT. **Figure S11.** Colony PCR verification of *farR* deletion in EDT△*argR*. **Figure S12.** Colony PCR verification of *ldh* deletion via pHA500sgRNA_*ldh*_ in EDT. **Figure S13.** Colony PCR verification of *ldh* deletion via pHA1000sgRNA_*ldh*_ in EDT. **Figure S14.** Colony PCR verification of 1-kb fragment deletion at the CGP3 locus in EDT. **Figure S15.** Colony PCR verification of 10-kb fragment deletion at the CGP3 locus in EDT. **Figure S16.** Colony PCR verification of 20-kb fragment deletion at the CGP3 locus in EDT. **Figure S17.** Colony PCR verification of *gfp* and *hom*-*thrB* insertion at the *upp* locus in EDT. **Figure S18.** Colony PCR verification of *gfp* insertion at the CGP1 locus in ET. **Figure S19.** Colony PCR verification of *gfp* insertion at the CGP2 locus in EDT. **Figure S20.** Colony PCR verification of *gfp* insertion at the CGP3 locus in EDT. **Figure S21.** Colony PCR verification of P_*tuf*_-*hom*-*thrB*, P_*tuf*_-*hom*-*thrB*-P_*glyA*_-*lysC*-*thrC* and P_*tuf*_-*trpEG*-P_*glyA*_- *trpDC*-P_*sod*_-*trpBA* insertions at the *upp* locus in EDT. **Figure S22.** Colony PCR verification of *lacZ* fragment insertion into the genomic locus between *cgl0900* and *cgl0901*. **Figure S23.** Colony PCR verification of *ldh* deletion in EDT. **Figure S24.** Colony PCR verification of *hdpA* deletion in EDT△*ldh*. **Figure S25.** Colony PCR verification of plug out of *cas9*–*recET* expression cassette in EDT△*ldh*△*hdpA*P_*hom*_-*pgk*. **Table S1.** Editing efficiencies using different genome editing methods in *C. glutamicum*. **Table S2.** Strains used in this study. **Table S3.** Plasmids used in this study. **Table S4.** Primers used in this study.

**Additional file 2:** Standard protocol of a RecET-assisted CRISPR-Cas9 genome editing in *Corynebacterium glutamicum*.

## Abbreviations

TCCRAS: toxin counter-selectable cassette regulated by an antitoxin switch
CRISPR: clustered regularly interspaced short palindromic repeat
sgRNA: single guide RNA
DSB: double-strand break
PAM: protospacer adjacent motif
NHEJ: nonhomologous end joining
HR: homologous recombination
ssDNA: single-stranded DNA
RBS: ribosome binding sequences
EDT: editable type
ssODN: single-stranded oligodeoxyribonucleotides
PCR: polymerase chain reaction
HA: homologous arm
UHA: upstream homologous arm
DHA: downstream homologous arm
qRT-PCR: quantitative reverse transcription PCR
PBS: phosphate-buffered saline
OD_600_: optical density at 600 nm
HPLC: high-pressure liquid chromatography
OPA: *O*-phthalaldehyde
VWD: variable wavelength detector
GCMS: gas chromatograph mass spectrometer
IPTG: isopropyl-β-d-thiogalactopyranoside
