# Supplementary material for: A RecET-assisted CRISPR–Cas9 genome editing in Corynebacterium glutamicum
Source: Microb Cell Fact. 2018 Apr 23;17:63. doi: 10.1186/s12934-018-0910-2 (PMC5913818; doi:10.1186/s12934-018-0910-2)
Supplement: Supplementary file 1 — Additional file 1: Figure S1. Colony PCR verification of cas9 integration. Figure S2. Colony PCR verification of upp deletion in WT::Phom-cas9. Figure S3. Colony PCR verification of upp deletion in WT::Ptuf-cas9. Figure S4. Colony PCR verification of upp deletion in WT::Ptuf-rbs1-cas9. Figure S5. Colony PCR verification of upp deletion in WT::Ptuf-rbs2-cas9. Figure S6. Colony PCR verification of recET integration. Figure S7. Colony PCR verification of upp deletion in WT::Ptuf-rbs2-cas9::Pprp-recET. Figure S8. Colony PCR verification of upp deletion in WT::Ptuf-rbs2-cas9::Pprp-rbs3-recET. Figure S9. Colony PCR verification of upp deletion in WT::Ptuf-rbs2-cas9::Pprp-rbs4-recET (EDT). Figure S10. Colony PCR verification of argR deletion in EDT. Figure S11. Colony PCR verification of farR deletion in EDT△argR. Figure S12. Colony PCR verification of ldh deletion via pHA500sgRNAldh in EDT. Figure S13. Colony PCR verification of ldh deletion via pHA1000sgRNAldh in EDT. Figure S14. Colony PCR verification of 1-kb fragment deletion at the CGP3 locus in EDT. Figure S15. Colony PCR verification of 10-kb fragment deletion at the CGP3 locus in EDT. Figure S16. Colony PCR verification of 20-kb fragment deletion at the CGP3 locus in EDT. Figure S17. Colony PCR verification of gfp and hom-thrB insertion at the upp locus in EDT. Figure S18. Colony PCR verification of gfp insertion at the CGP1 locus in ET. Figure S19. Colony PCR verification of gfp insertion at the CGP2 locus in EDT. Figure S20. Colony PCR verification of gfp insertion at the CGP3 locus in EDT. Figure S21. Colony PCR verification of Ptuf-hom-thrB, Ptuf-hom-thrB-PglyA-lysC-thrC and Ptuf-trpEG-PglyA- trpDC-Psod-trpBA insertions at the upp locus in EDT. Figure S22. Colony PCR verification of lacZ fragment insertion into the genomic locus between cgl0900 and cgl0901. Figure S23. Colony PCR verification of ldh deletion in EDT. Figure S24. Colony PCR verification of hdpA deletion in EDT△ldh. Figure S25. Colony PCR verific [file 12934_2018_910_MOESM1_ESM.docx]

**Additional file 1**

**A RecET-assisted CRISPR-Cas9 genome editing in *Corynebacterium glutamicum***

Bo Wang^1, 2#^, Qitiao Hu^1, 2#^, Yu Zhang^1, 2^, Ruilin Shi^3^, Xin Chai^3^, Zhe Liu^1, 2^, Xiuling Shang^1^, Yun Zhang^1^*, Tingyi Wen^1, 3, 4^*

^1^CAS Key Laboratory of Pathogenic Microbiology and Immunology, Institute of Microbiology, Chinese Academy of Sciences, Beijing 100101, China

^2^University of Chinese Academy of Sciences, Beijing 100049, China

^3^Beijing Zhongke Eppen Biotech Co., Ltd，Beijing 100085, China

^4^Savaid Medical School, University of Chinese Academy of Sciences, Beijing 100049, China

^#^B.W. and Q.H. contributed equally to this work.

*Correspondence: wenty@im.ac.cn; [zhangyun@im.ac.cn](mailto:zhangyun@im.ac.cn)

**Figure S1** Colony PCR verification of *cas9* integration.


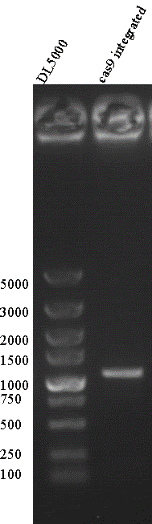


**Figure S2** Colony PCR verification of *upp* deletion in WT::P*_hom_*-*cas9*.


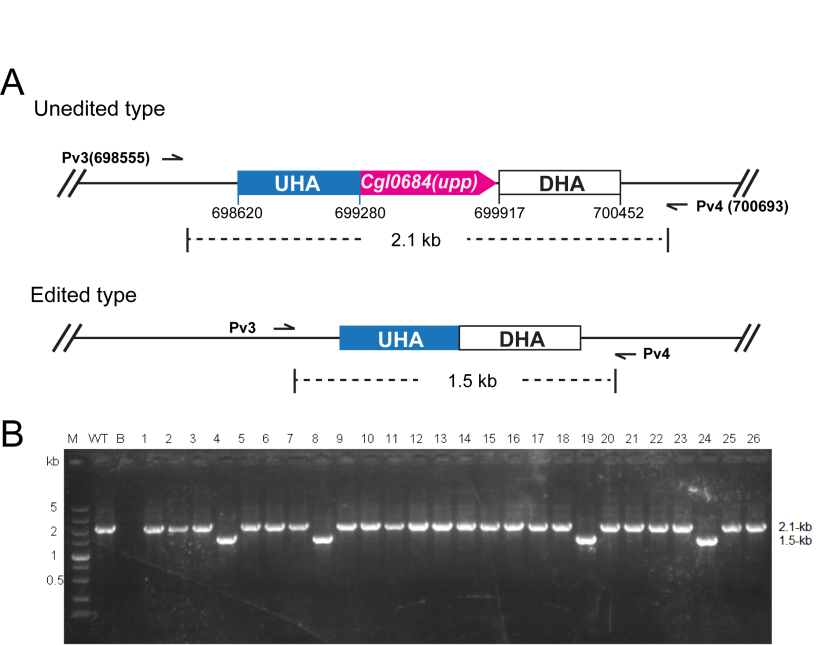


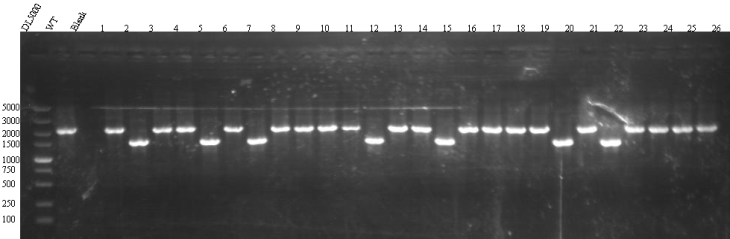

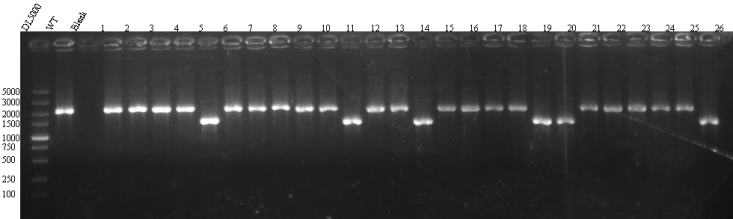


**Figure S3** Colony PCR verification of *upp* deletion in WT::P*_tuf_*-*cas9*.


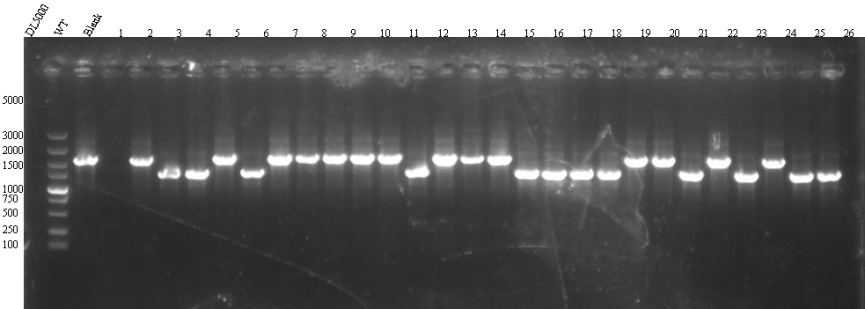

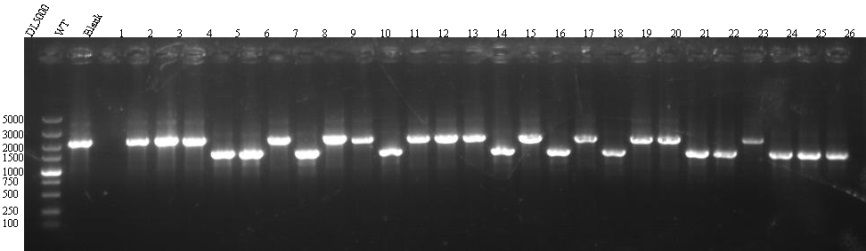

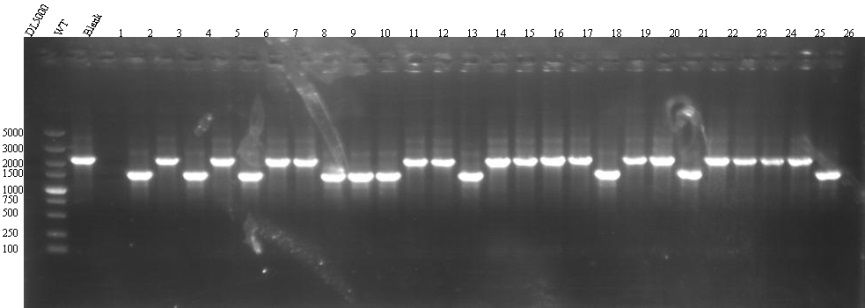


**Figure S4** Colony PCR verification of *upp* deletion in WT::P*_tuf_*-rbs1-*cas9*.

**
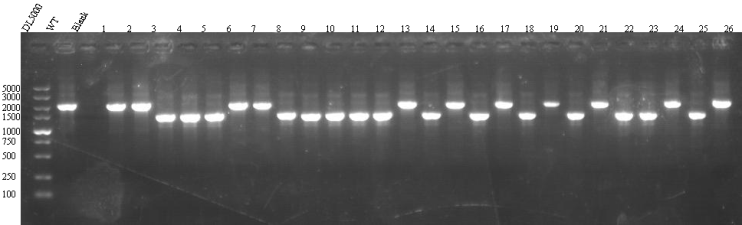

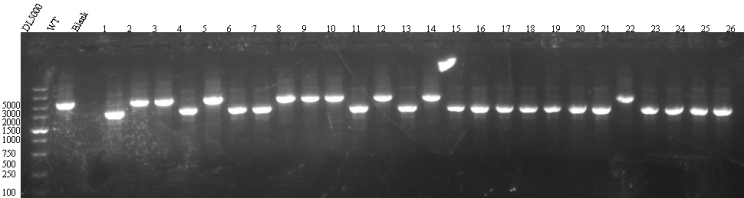

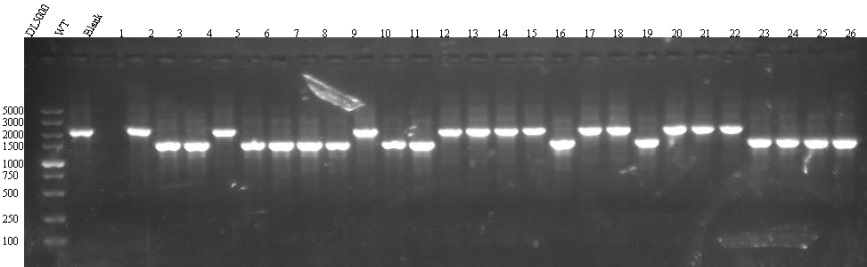
**

**Figure S5** Colony PCR verification of *upp* deletion in WT::P*_tuf_*-rbs2-*cas9*.


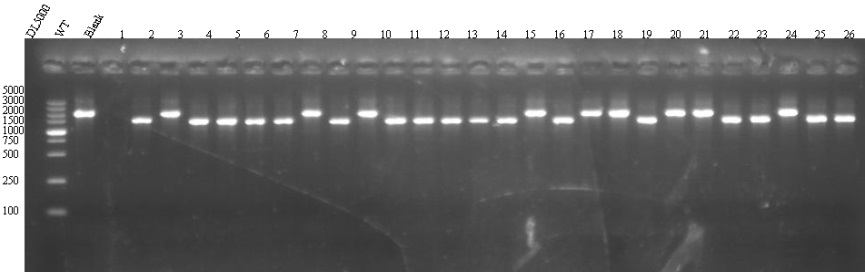

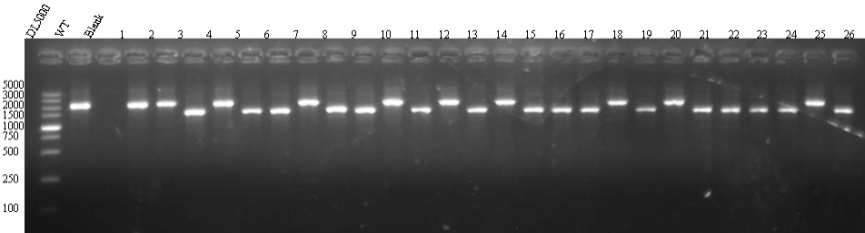

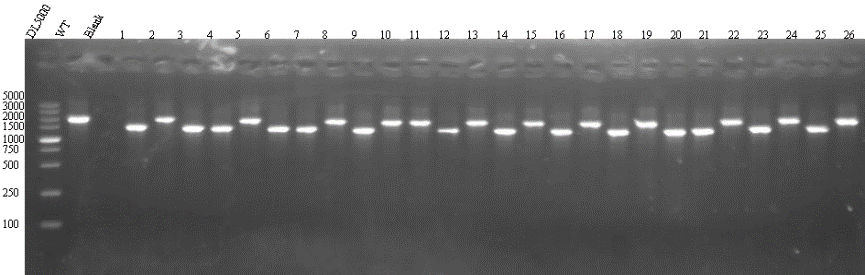


**Figure S6** Colony PCR verification of *recET* integration.


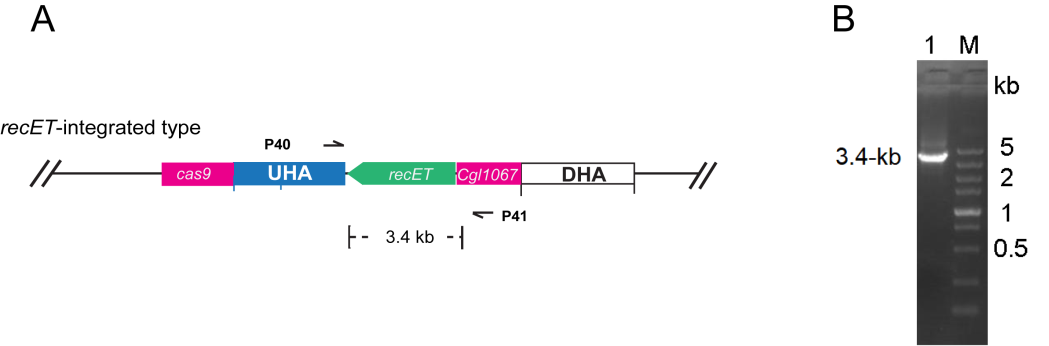


**Figure S7** Colony PCR verification of *upp* deletion in WT::P*_tuf_*-rbs2-*cas9*::P*_prp_*-*recET*.


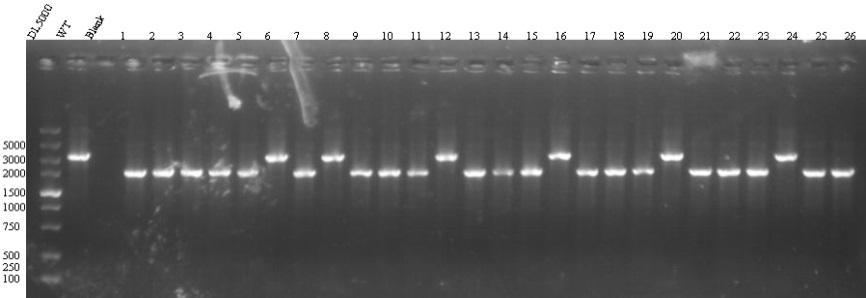

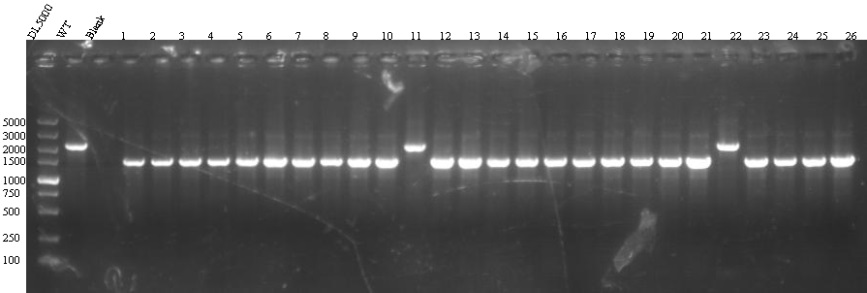

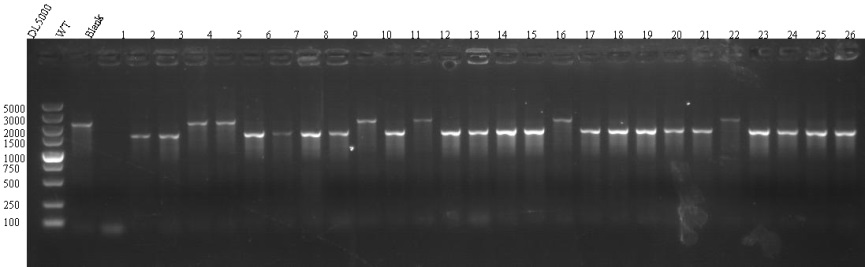


**Figure S8** Colony PCR verification of *upp* deletion in WT::P*_tuf_*-rbs2-*cas9*::P*_prp_*-rbs3-*recET*.


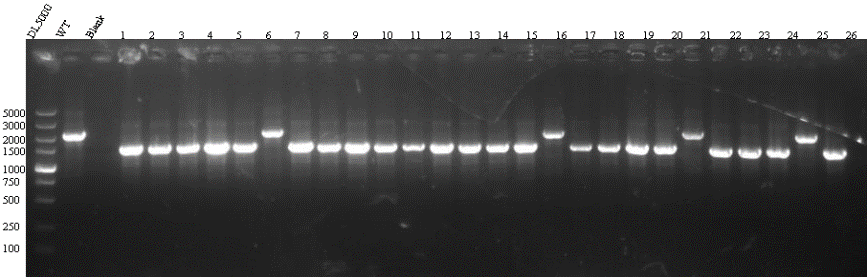

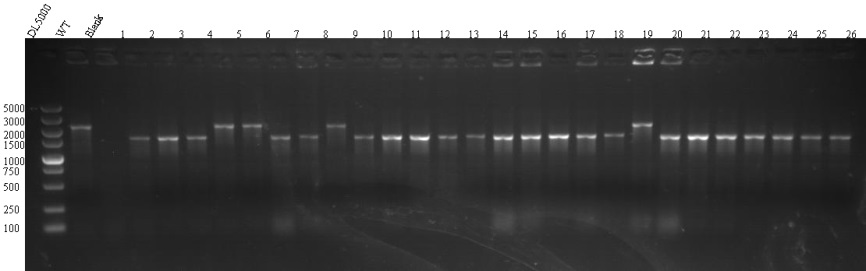

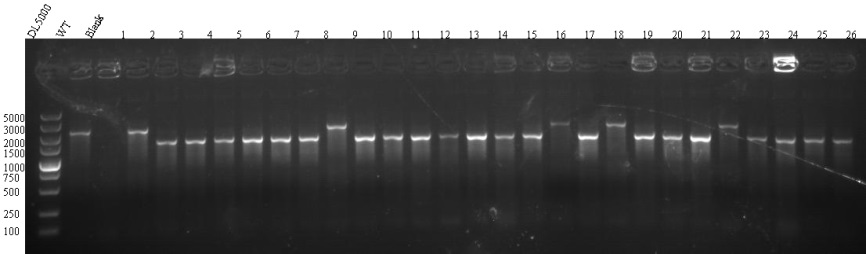


**Figure S9** Colony PCR verification of *upp* deletion in WT::P*_tuf_*-rbs2-*cas9*::P*_prp_*-rbs4-*recET* (EDT).


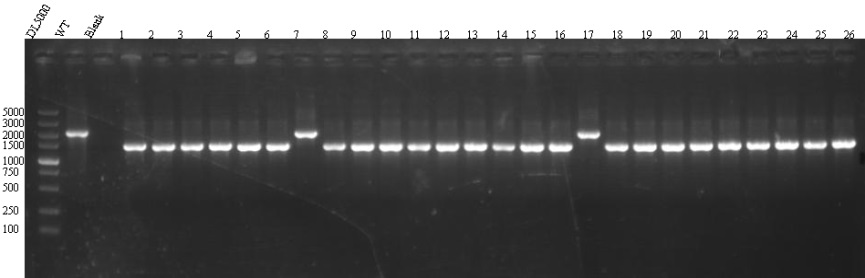

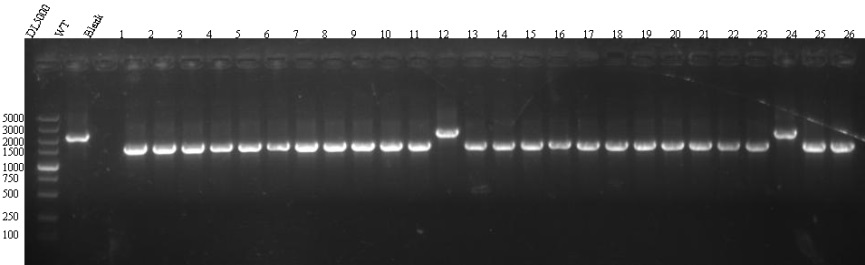

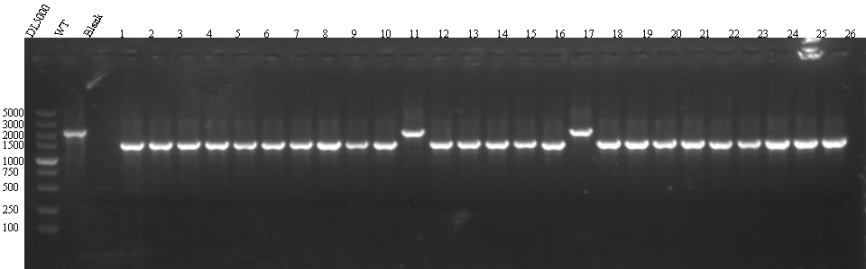


**Figure S10** Colony PCR verification of *argR* deletion in EDT.


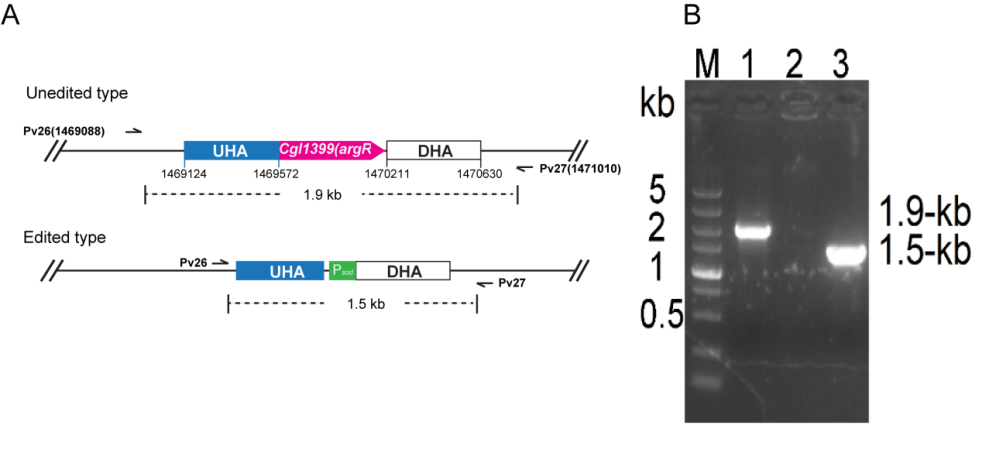


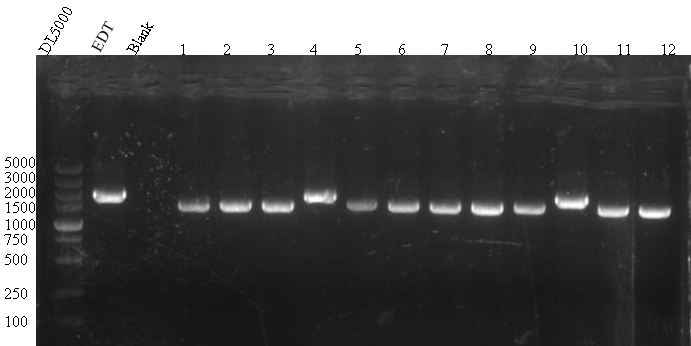


**Figure S11** Colony PCR verification of *farR* deletion in EDT△*argR*.


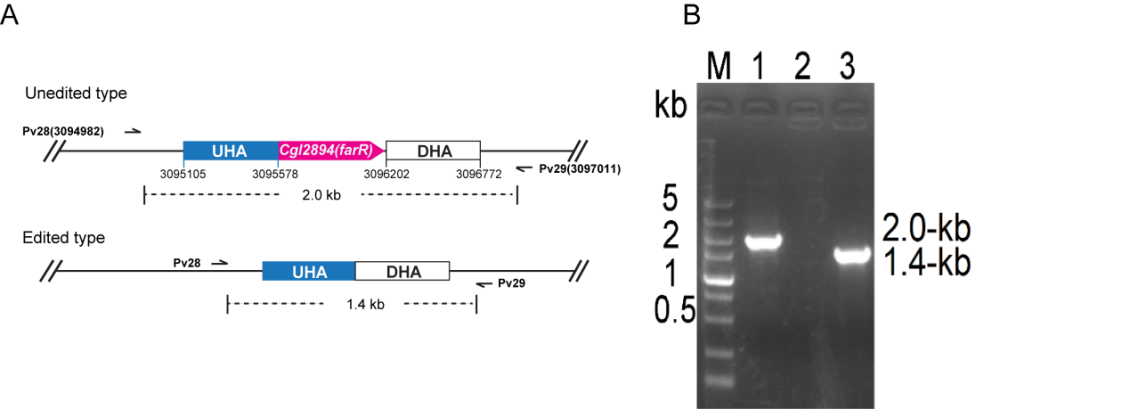


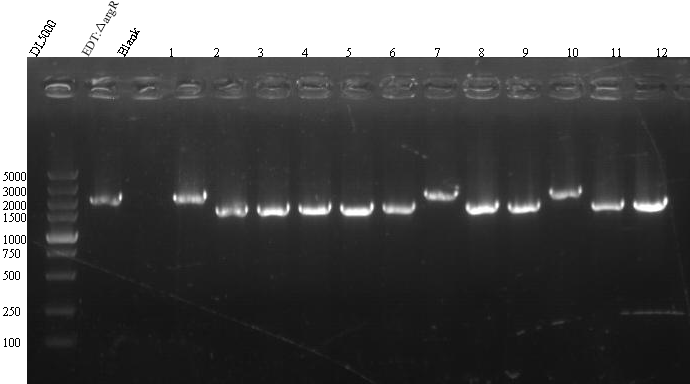


**Figure S12** Colony PCR verification of *ldh* deletion *via* pHA500sgRNA*_ldh_* in EDT.


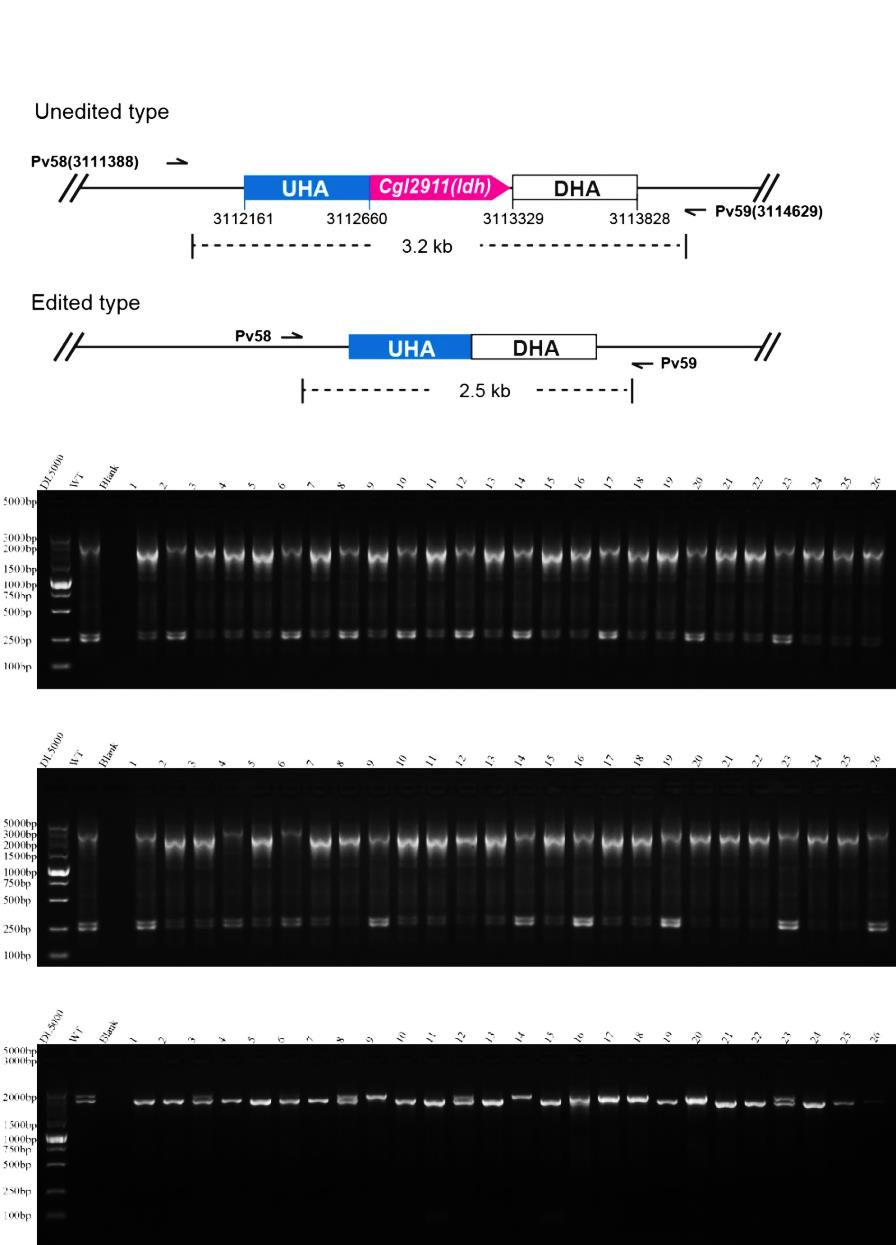


**Figure S13** Colony PCR verification of *ldh* deletion *via* pHA1000sgRNA*_ldh_* in EDT.


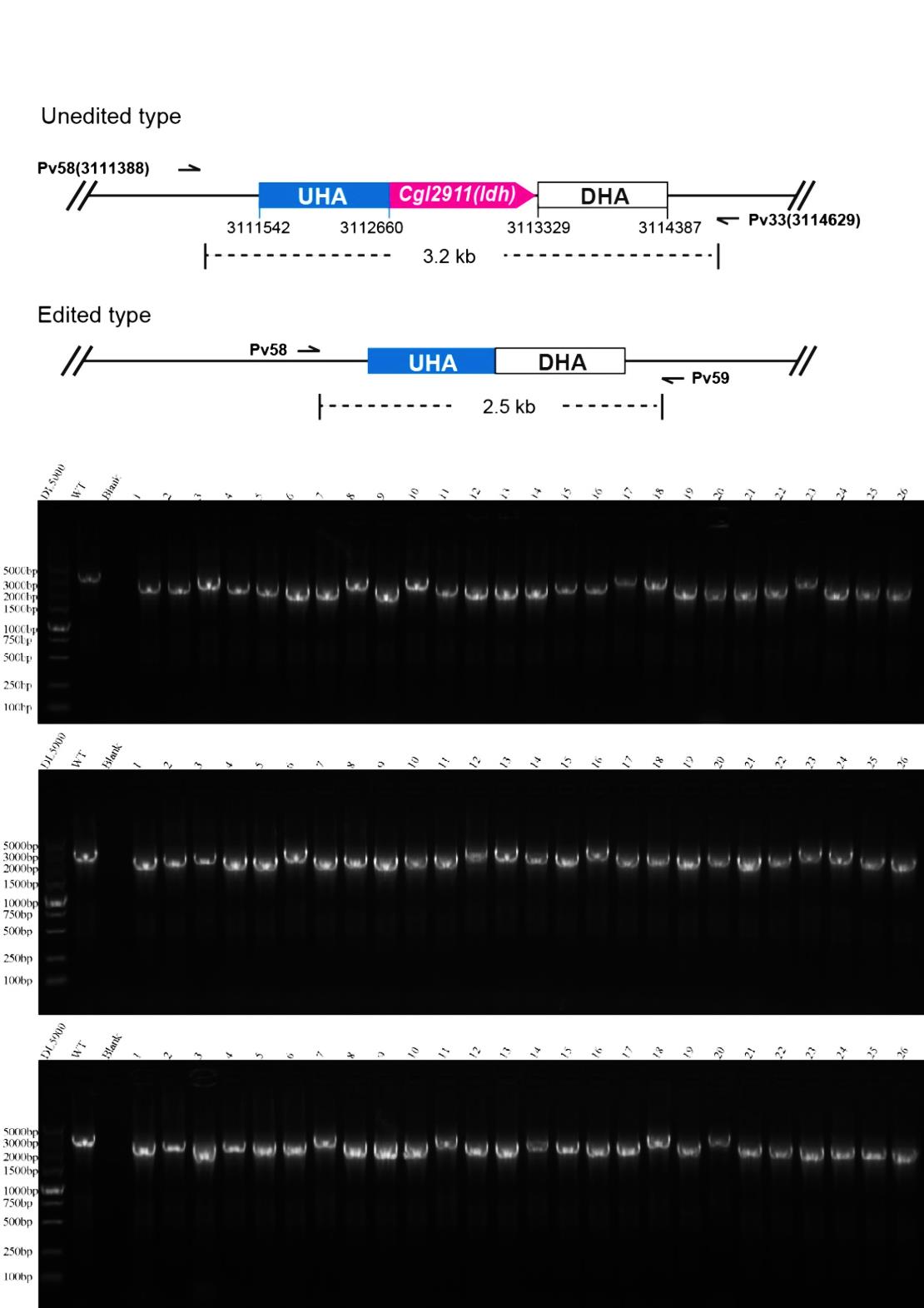


**Figure S14** Colony PCR verification of 1-kb fragment deletion at the CGP3 locus in EDT.


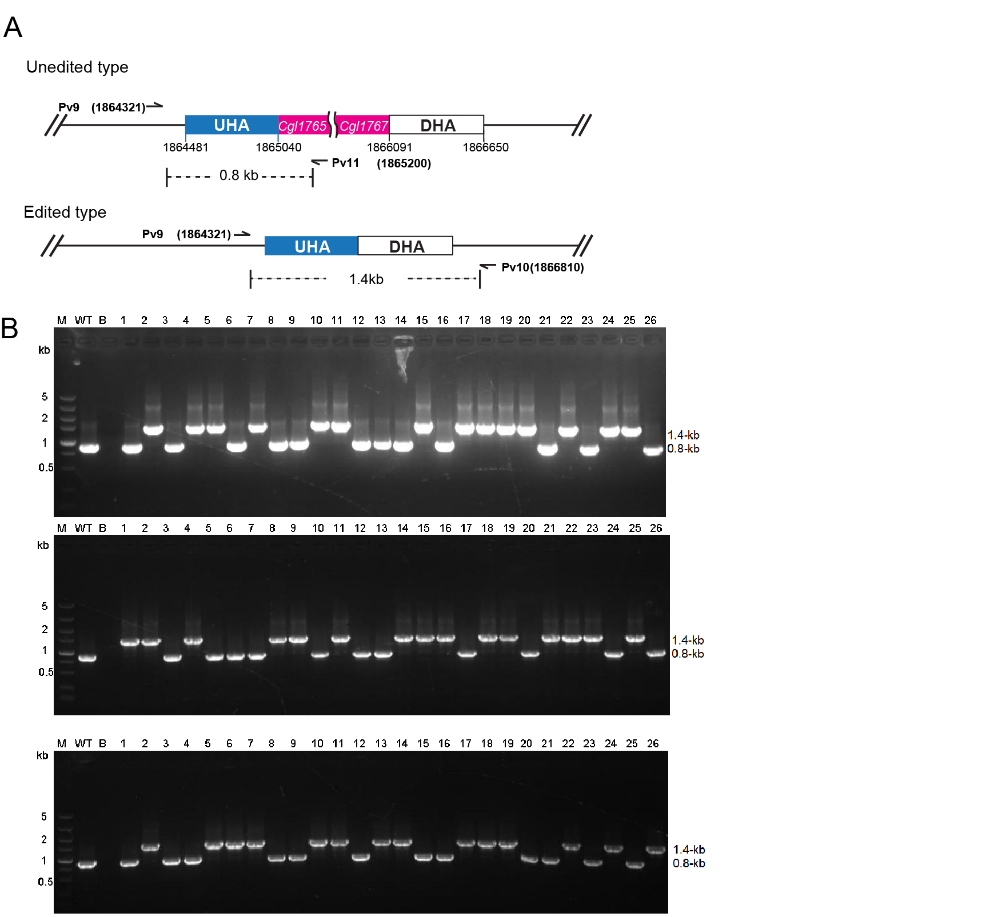


**Figure S15** Colony PCR verification of 10-kb fragment deletion at the CGP3 locus in EDT.


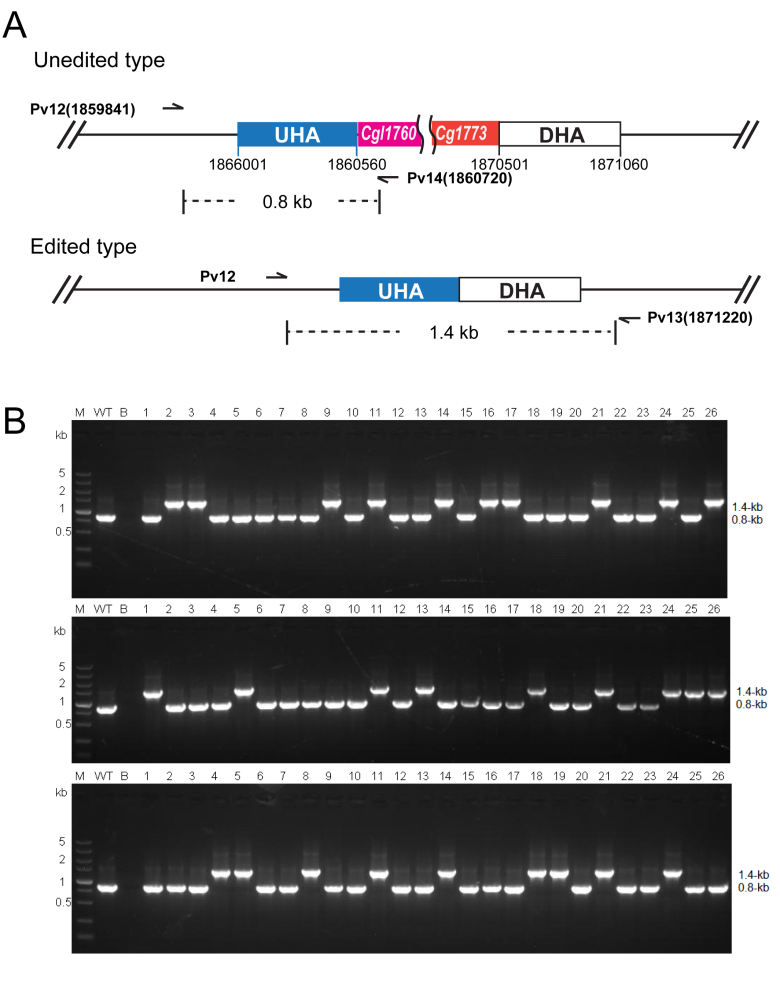


**Figure S16** Colony PCR verification of 20-kb fragment deletion at the CGP3 locus in EDT.


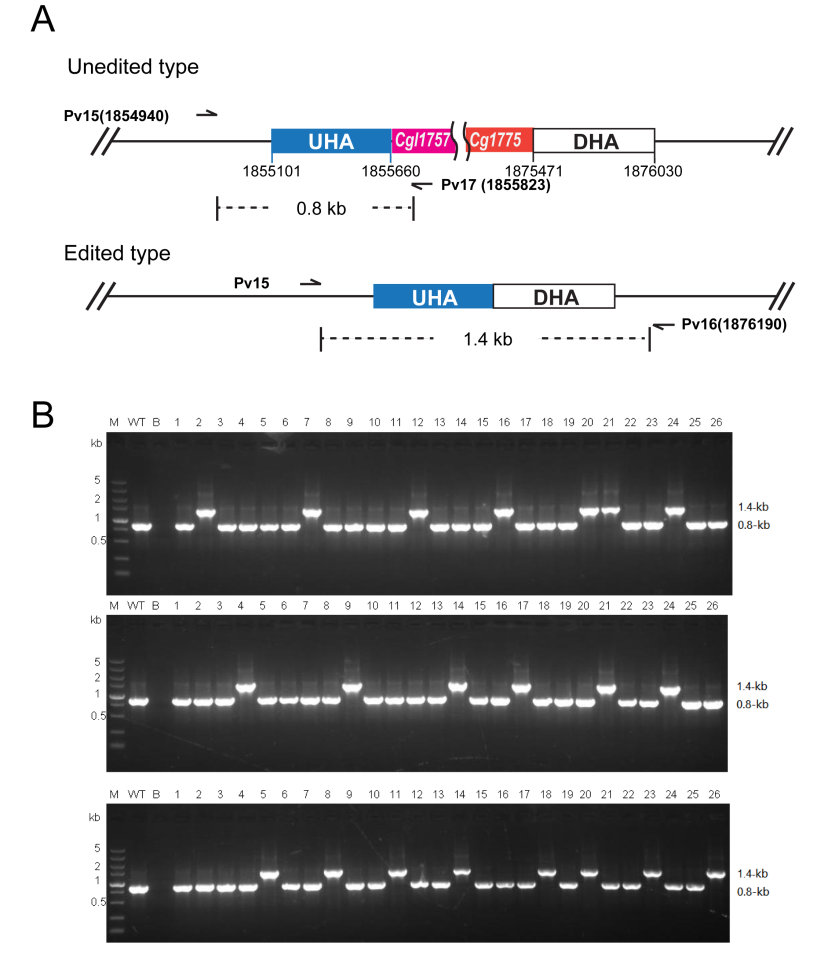


**Figure S17** Colony PCR verification of *gfp* insertion at the *upp* locus in EDT.


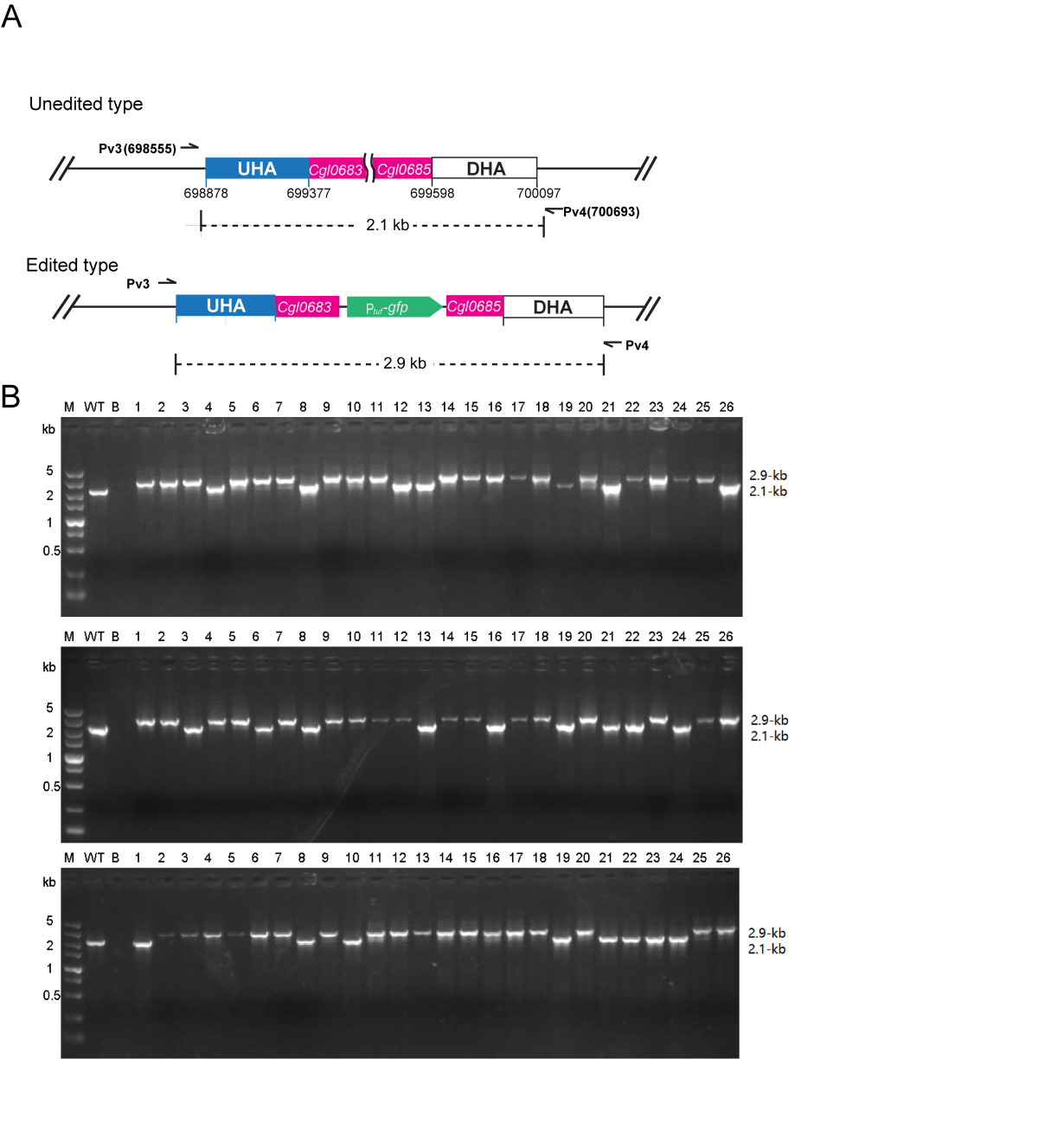


**Figure S18** Colony PCR verification of *gfp* insertion at the CGP1 locus in EDT.


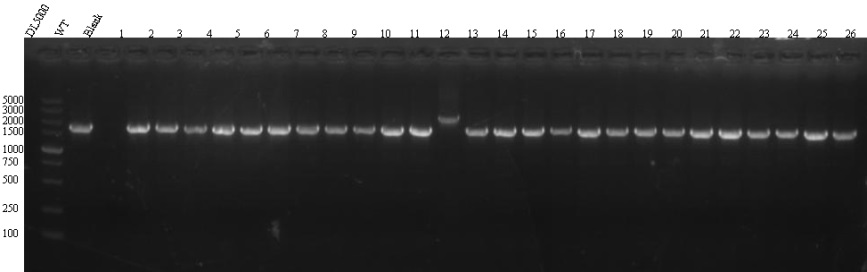

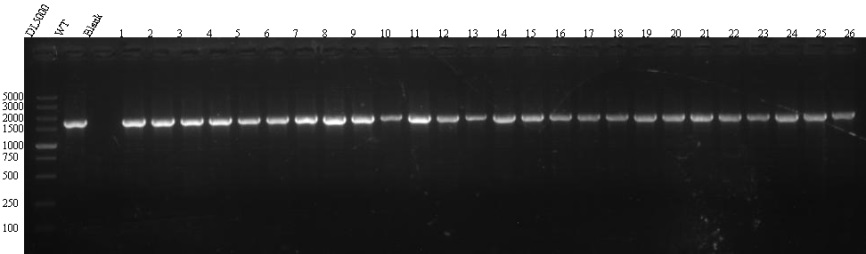

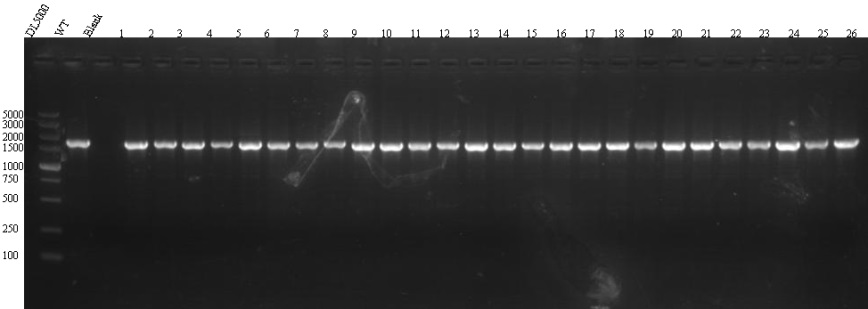


**Figure S19** Colony PCR verification of *gfp* insertion at the CGP2 locus in EDT.


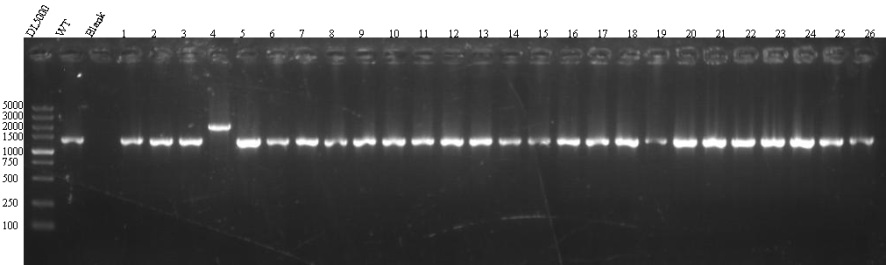

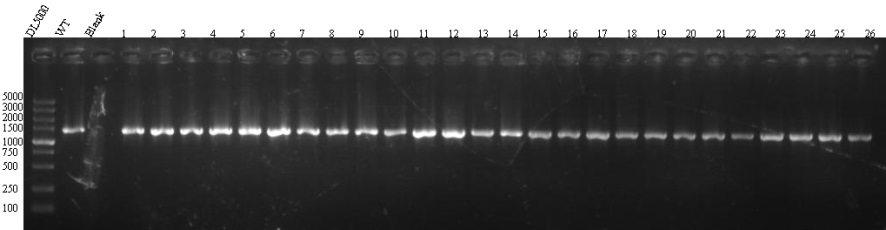

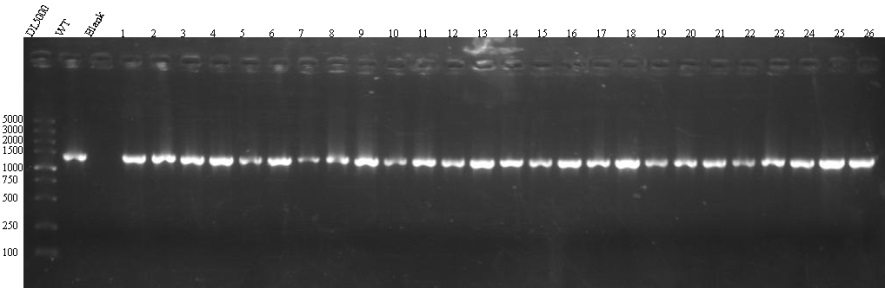


**Figure S20** Colony PCR verification of *gfp* insertion at the CGP3 locus in EDT.


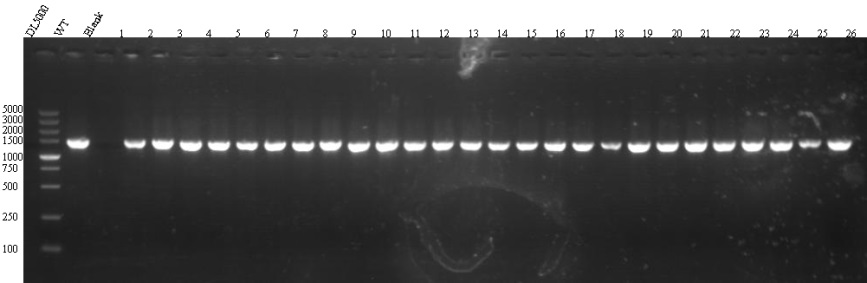

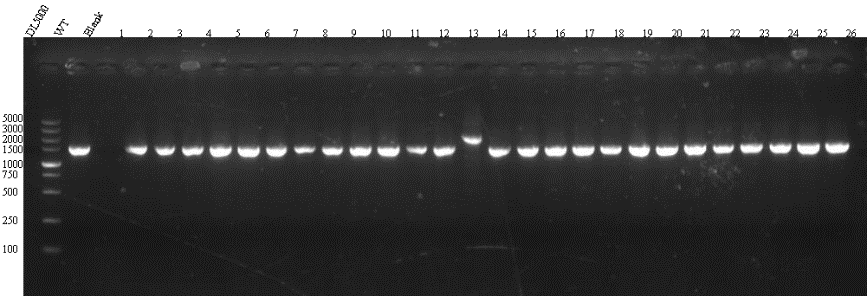

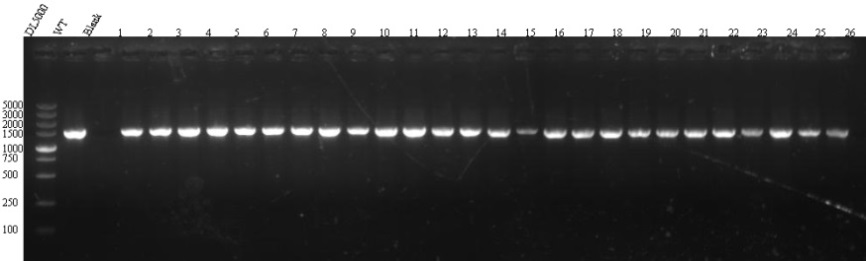


**Figure S21** Colony PCR verification of P*_tuf_*-*hom*-*thrB*, P*_tuf_*-*hom*-*thrB-*P*_glyA_-lysC-thrC* and P*_tuf_*-*trpEG*-P*_glyA_*-*trpDC-*P*_sod_*-*trpBA* insertions at the *upp* locus in EDT. A. Colony PCR verification of P*_tuf_*-*hom*-*thrB* insertion. B. Colony PCR verification of P*_tuf_*-*hom*-*thrB-*P*_glyA_-lysC-thrC* insertion. C. Colony PCR verification of P*_tuf_*-*trpEG*-P*_glyA_*-*trpDC-*P*_sod_*-*trpBA* insertion.

**
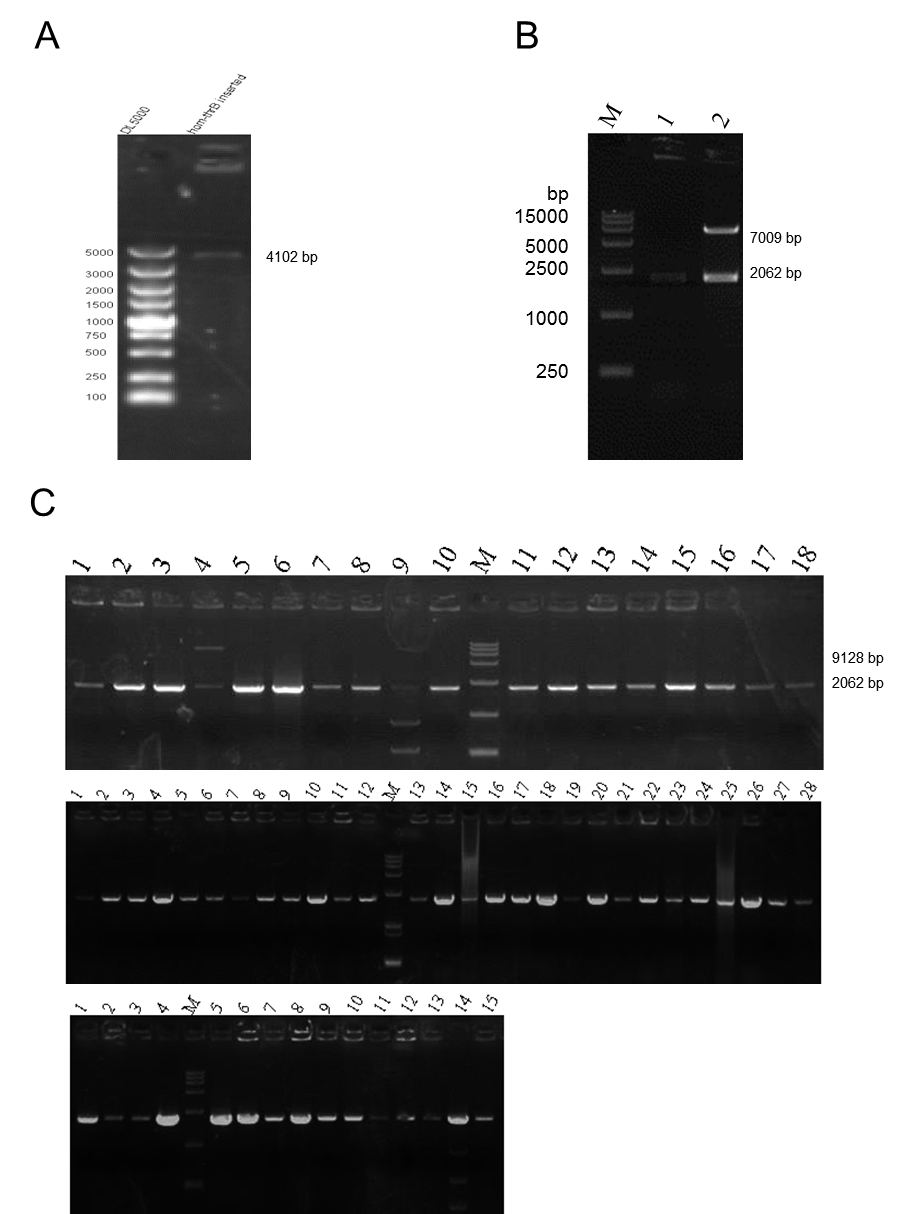
**

**Figure S22** Colony PCR verification of *lacZ* fragment insertion into the genomic locus between *cgl0900* and *cgl0901*. A. Colony PCR verification of *lacZ* fragment insertion using p*lacZ*HAsgRNA*_cgl0900-cgl0901_*_-1_. B. Colony PCR verification of *lacZ* fragment insertion using p*lacZ*HAsgRNA*_cgl0900-cgl0901_*-_2_.

**
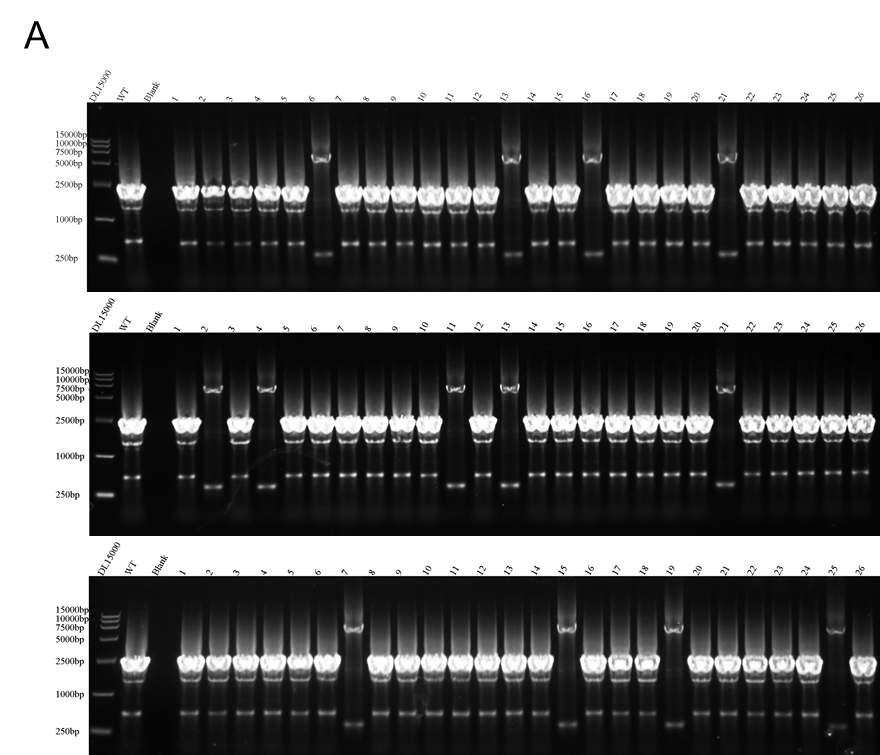
**

**
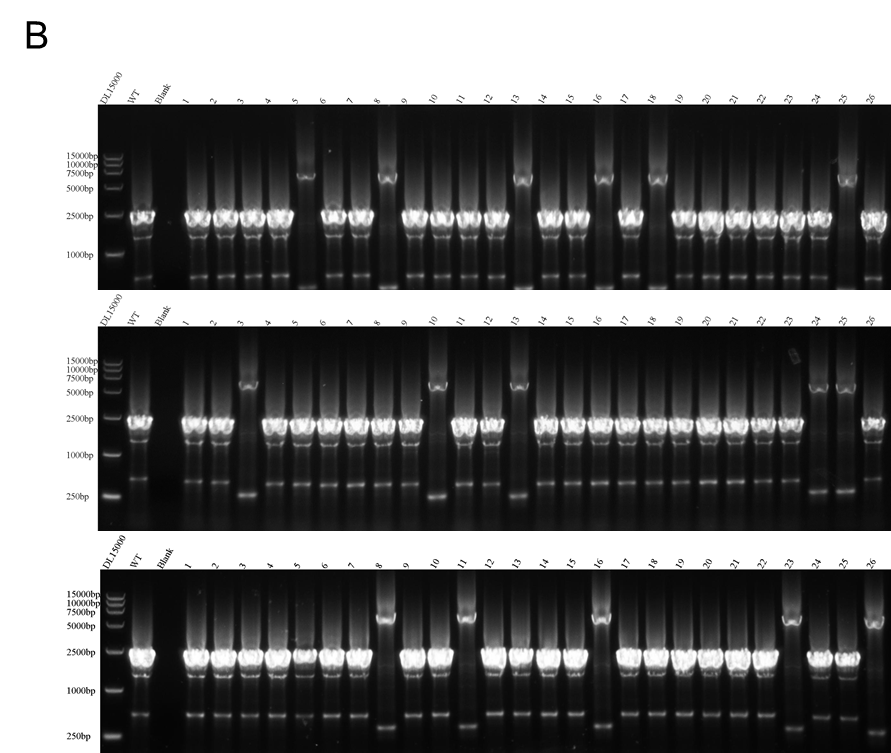
**

**Figure S23** Colony PCR verification of *ldh* deletion in EDT.


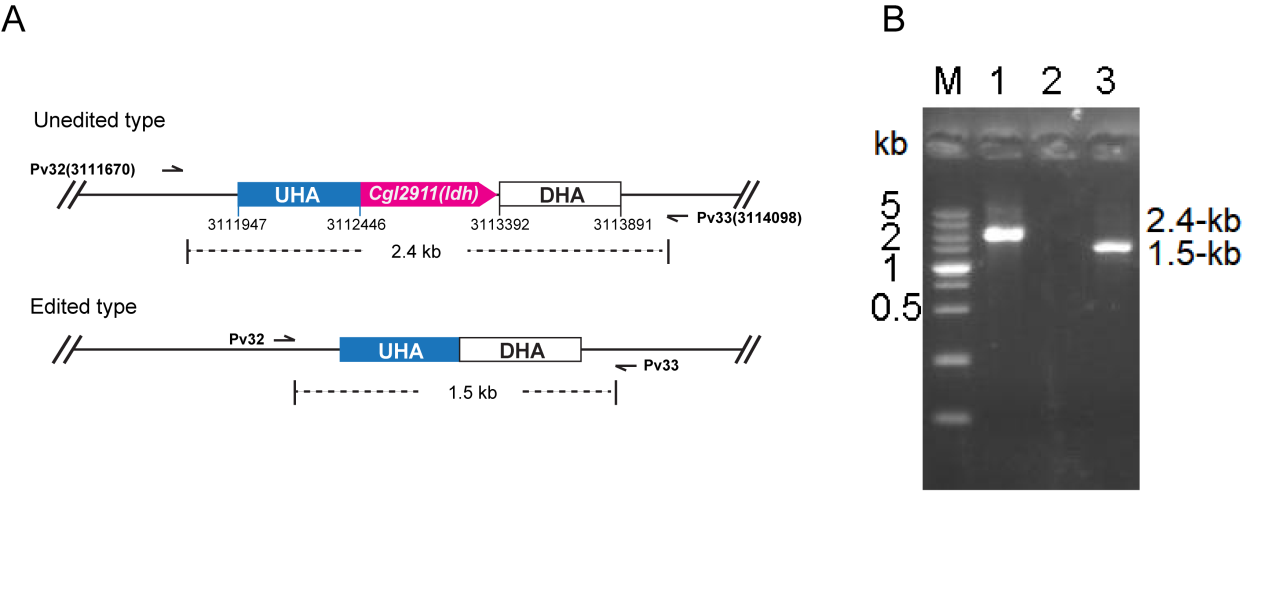


**Figure S24** Colony PCR verification of *hdpA* deletion in EDT△*ldh*.


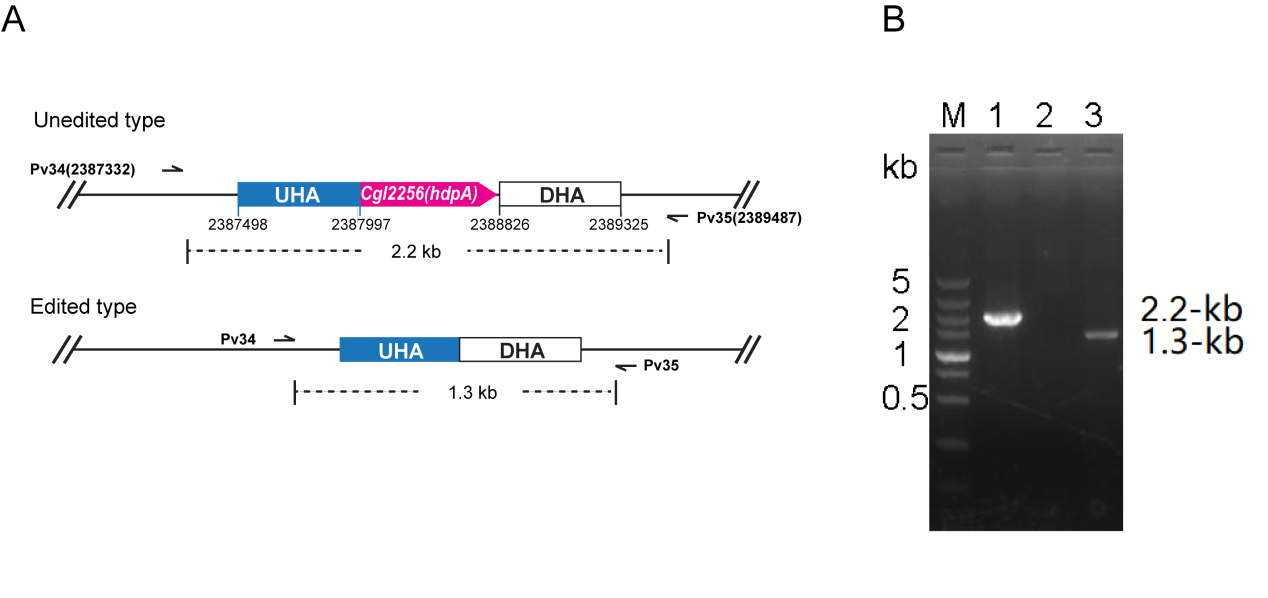


**Figure S25** Colony PCR verification of plug out of *cas9-recET* expression cassette in EDT△*ldh*△*hdpA*P*_hom_*-*pgk*.


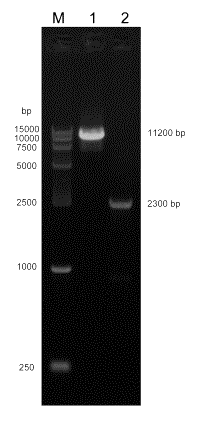


**Table S1** Editing efficiencies using different genome editing methods in *C. glutamicum*. [1-4]

| Locus | Fragment length (bp) |  | Editing Efficiency % | | | | | | | | | | | |
| --- | --- | --- | --- | --- | --- | --- | --- | --- | --- | --- | --- | --- | --- | --- |
|  |  |  | This work | | 2017, Microb Cell Fact [3] | | 2017, Microb Cell Fact [4] | | 2017, Metab Eng [2] | | 2017, Nat Commun [1] | | | pK18*mobsacB* [5] |
| **Deletion** |  |  |  | |  | |  | |  | |  | | |  |
| *crtYf* | 50 |  | - | | - | | - | | - | | 15 | | | - |
| *gabT* | 100 |  | - | | - | | - | | 100 | | - | | | - |
| *gabP* | 150 |  | - | | - | | - | | 87.5 | | - | | | - |
| N*Cgl1221* | 250 |  | - | | - | | - | | 55.17 | | - | | | - |
| *gabT* | 300 |  | - | | - | | - | | 80 | | - | | | - |
| *porB* | 377 |  | - | | 100 | | - | | - | | - | | | - |
| *argR* | 400 |  | - | | - | | - | | 23.81 | | - | | | - |
| N*Cgl1221* | 400 |  | - | | - | | - | | 34.48 | | - | | | - |
| *crtYf* | 500 |  | - | | - | | - | | - | | 0 | | | - |
| *argR* | 516 |  | 83.33 | | - | | - | | - | | - | | | - |
| *mepA* | 576 |  | - | | 0-100 | | - | | - | | - | | | - |
| *upp* | 636 |  | 92.31 | | - | | - | | - | | - | | | 43.59 |
| *crtYf* | 705 |  | - | | - | | - | | - | | 15 | | | - |
| *farR* | 717 |  | 75 | | - | | - | | - | | - | | | - |
| *hdpA* | 828 |  | 66.67 | | - | | - | | - | | - | | | - |
| *ldh* (~500 HA) | 668 |  | 65.38 | | - | | 50.0 | | - | | - | | | - |
| *ldh* (~1000 HA) | 668 |  | 78.21 | | - | | 60.0 | | - | | - | | | - |
| *ldh* | 945 |  | 50 | | - | | - | | - | | - | | | - |
| CGP3 | 1000 |  | 55.13 | | - | | - | | - | | - | | | - |
| NCgl0991 | 1260 |  | - | | 26.67 | | - | | - | | - | | | - |
| *clpX* | 1281 |  | - | | 31.25 | | - | | - | | - | | | - |
| *cg0716* to *cg0723* | 7500 |  | - | | - | | - | | - | | 10 | | | - |
| Cgl1775 | 8083 |  | - | | - | | 40 | | - | | - | | | - |
| CGP3 | 10000 |  | 35.9 | | - | | - | | - | | - | | | - |
| CGP3 | 20000 |  | 26.92 | | - | | - | | - | | - | | | - |
| **Insertion** |  |  |  | |  | |  | |  | |  | | |  |
| *gfp* | 720 |  | 69.23 | | 66.67 | | - | | - | | - | | | - |
| *rfp* | 781 |  | - | | - | | 25 | | - | | - | | | - |
| P*_tuf_-hom-thrB* | 2.5 k |  | 7.14 | | - | | - | | - | | - | | | - |
| *lacZ* fragment | 3.6 k |  | 16.67 / 20.51 | | - | | 50 | | - | | - | | | - |
| P*_tuf_-hom-thrB-*P*_glyA_-lysC-thrC* | 5.7 k |  | 2.22 | | - | | - | | - | | - | | | - |
| P*_tuf_*-*trpEG*-P*_glyA_*-*trpDC-*P*_sod_*-*trpBA* | 7.5 k |  | 1.64 | | - | | - | | - | | - | | | - |
| **Point mutation** |  |  | |  | |  | |  | |  | |  |  | |
| *pgi* |  |  | 95 | | - | | - | | - | | - | | | - |
| *argB* |  |  | 77 | | - | | - | | - | | - | | | - |
| *crtYf* |  |  | - | | - | | - | | - | | 86-100 | | | - |
| *porB* |  |  | - | | 100 | | - | | - | | - | | | - |
| *rfp* |  |  | - | | - | | 86.7 | | - | | - | | | - |
| *rpsL* |  |  | - | | - | | 70 | | - | | - | | | - |
| **Replacement** |  |  |  | |  | |  | |  | |  | | |  |
| ΔP*_pgk_*::P*_hom_* | Δ200 bp :: 170 bp |  | 66.67 | | - | | - | | - | | - | | | - |
| ΔP*_pgk_*::P*_dapA_* | Δ200 bp :: 200 bp |  | 83.33 | | - | | - | | - | | - | | | - |
| Δ*lysE*::*cadA* | Δ0.7 kbp :: 2.1 kbp |  | - | | - | | - | | ~5 | | - | | | - |
| Δ*crtYf*::*tdc* | Δ0.7 kbp :: 1 kbp |  | - | | - | | - | | - | | 38.5 | | | - |

**Table S2** Strains used in this study.

| Strain | Genotype | Source |
| --- | --- | --- |
| EC135 | *E. coli* TOP10△*dam*△*dcm* | [6] |
| *Corynebacterium glutamicum* ATCC 13032 | Wild Type | American Type and Culture Collection |
| *Corynebacterium pekinense* CGMCC 1.563 | Wild Type | China General Microbiological Culture Collection Center |
| WT::P*_hom_*-*cas9* | WT::P*_hom_*-*cas9* | This study |
| WT::P*_tuf_*-*cas9* | WT::P*_tuf_*-*cas9* | This study |
| WT::P*_tuf_*-rbs1-*cas9* | WT::P*_tuf_*-*cas9* with optimized RBS1 for *cas9*^a^ | This study |
| WT::P*_tuf_*-rbs2-*cas9* | WT::P*_tuf_*-*cas9* with optimized RBS2 for *cas9*^b^ | This study |
| WT::P*_tuf_*- rbs2-*cas9*::P*_prp_*-*recET* | WT::P*_tuf_*-rbs2-*cas9*::P*_prp_*-*recET* |  |
| WT::P*_tuf_*-rbs2-*cas9*::P*_prp_*-rbs3-*recET* | WT::P*_tuf_*-rbs2-*cas9*::P*_prp_*-*recET* with optimized RBS3 for *recET*^c^ | This study |
| WT::P*_tuf_*-rbs2-*cas9*::P*_prp_*-rbs4-*recET* (EDT for short) | WT::P*_tuf_*-rbs2-*cas9*::P*_prp_*-*recET* with optimized RBS4 for *recET*^d^ | This study |
| EDT△*argR* | EDT△*argR* | This study |
| EDT△*argR*△*farR* | EDT△*argR*△*farR* | This study |
| EDT△*argR*△*farR* *argB** | EDT△*argR*△*farR* *argB** | This study |
| PT | EDT/pXMJ19-pXMJ19*-mgsA-gldA-yqhD*^e^ | This study |
| PT Δ*ldh* Δ*hdpA* | EDT Δ*ldh* Δ*hdpA*/pXMJ19-pXMJ19*-mgsA-gldA-yqhD* | This study |
| PT Δ*ldh* Δ*hdpA* P*_hom_*-*pgk* | EDT Δ*ldh* Δ*hdpA* P*_hom_*-*pgk*/ pXMJ19-pXMJ19*-mgsA-gldA-yqhD* | This study |
| PT Δ*ldh* Δ*hdpA* P*_dapA_*-*pgk* | EDT Δ*ldh* Δ*hdpA* P*_dapA_*-*pgk*/ pXMJ19-pXMJ19*-mgsA-gldA-yqhD* | This study |
| WTΔ*ldh* Δ*hdpA* P*_dapA_*-*pgk* | EDT Δ*ldh* Δ*hdpA* P*_hom_*-*pgk* Δ*cas9* Δ*recET* | This study |

1. The sequence of RBS1 for *cas9* is AGCTATACAGACCCCCCACATACAACAACAAGGAGACACGTT.
2. The sequence of RBS2 for *cas9* is AGCTACTACAGACCCCCTACAAACAACAAGAAGGAGACGGATT.
3. The sequence of RBS3 for *recET* is CGGCCATTAGGGGGAAGCTTAAAGGAGGAAACTTT.
4. The sequence of RBS4 for *recET* is GACCAAAACATTACAAGCTTAAAGGAGGAAACTTT.
5. The sequences of RBS for *mgsA*, *gldA* and *yqhD* are GCCCTCGTAAGCTAGAGGAGGTTAAC, CGCTATTCCGCAAGGACAACAAACAGAAAGGAGGTAAAAC and CCAGTACACAGAAATACAAAGAGGAGGTAACTA

**Table S3** Plasmids used in this study.

| Plasmid | Description | Resistance marker | Source |
| --- | --- | --- | --- |
| pK18*mobsacB* | Integrative vector | Kanamycin | [5] |
| pXMJ19 | Expression vector in *C. glutamicum* | Chloramphenicol | [7] |
| pTargetF | Template for the amplification of the sgRNA scaffold | Spectinomycin | Addgene [8] |
| pIN-P*_hom_*-*cas9* | Integrate P*_hom_*-*cas9* to the genome of *C. glutamicum* | Kanamycin | This study |
| pIN-P*_tuf_*-*cas9* | Integrate P*_tuf_*-*cas9* to the genome of *C. glutamicum* | Kanamycin | This study |
| pIN-P*_tuf_*-RBS1-*cas9* | Integrate P*_tuf_*-RBS1-*cas9* to the genome of *C. glutamicum* | Kanamycin | This study |
| pIN-P*_tuf_*-RBS2-*cas9* | Integrate P*_tuf_*-RBS2-*cas9* to the genome of *C. glutamicum* | Kanamycin | This study |
| pIN-P*_prp_*-*recET* | Integrate P*_prp_*-*recET* to the genome of *C. glutamicum* | Kanamycin | This study |
| pIN-P*_prp_*-RBS1-*recET* | Integrate P*_prp_*-RBS1-*recET* to the genome of *C. glutamicum* | Kanamycin | This study |
| pIN-P*_prp_*-RBS2-*recET* | Integrate P*_prp_*-RBS2-*recET* to the genome of *C. glutamicum* | Kanamycin | This study |
| pHAsgRNA*_upp_* | Knock out *upp* | Chloramphenicol | This study |
| pHAsgRNA*_nc_* | Homology arm of *upp* with invalid sgRNA | Chloramphenicol | This study |
| pHA500sgRNA*_ldh_* | 500 bp homology arm flanking *ldh* with sgRNA | Chloramphenicol | This study |
| pHA1000sgRNA*_ldh_* | ~1 kb homology arm flanking *ldh* with sgRNA | Chloramphenicol | This study |
| pHAsgRNA_ΔCGP3-1kb_ | Knock out 1-kb fragment of CGP3 | Chloramphenicol | This study |
| pHAsgRNA_ΔCGP3-10kb_ | Knock out 10-kb fragment of CGP3 | Chloramphenicol | This study |
| pHAsgRNA_ΔCGP3-20kb_ | Knock out 20-kb fragment of CGP3 | Chloramphenicol | This study |
| p*gfp*HAsgRNA*_upp_* | Insert *gfp* into *upp* | Chloramphenicol | This study |
| p*hom-thrB*HAsgRNA*_upp_* | Insert *hom-thrB* into *upp* | Chloramphenicol | This study |
| p*hom-thrB-lysC-thrC*HAsgRNA*_upp_* | Insert *thrB-lysC-thrC* into *upp* | Chloramphenicol | This study |
| p*trpEGDCBA*HAsgRNA*_upp_* | Insert P*_tuf_-trpEG-*P*_glyA_-trpDC-*P*_sod_-trpBA* into *upp* | Chloramphenicol | This study |
| p*lacZ*HAsgRNA*_cgl0900-cgl0901_*_-1_ | Insert *lacZ* fragment (3626 bp) into the genomic locus between *cgl0900* and *cgl0901* using the same gRNA as reported previously [4] . | Chloramphenicol | This study |
| p*lacZ*HAsgRNA *_cgl0900-cgl0901_*_-2_ | Insert *lacZ* fragment (3626 bp) into the genomic locus between *cgl0900* and *cgl0901* using a gRNA different from a previous report [4]. | Chloramphenicol | This study |
| p*gfp*HAsgRNA_CGP1_ | Insert *gfp* into CGP1 | Chloramphenicol | This study |
| p*gfp*HAsgRNA_CGP2_ | Insert *gfp* into CGP2 | Chloramphenicol | This study |
| p*gfp*HAsgRNA_CGP3_ | Insert *gfp* into CGP3 | Chloramphenicol | This study |
| pHAsgRNA*_pgi_*_1_ | Mutate the start codon of *pgi* | Chloramphenicol | This study |
| pHAsgRNA*_pgi_*_2_ | Mutate the start codon and PAM of *pgi* | Chloramphenicol | This study |
| pHAsgRNA*_argR_* | Knock out *argR* | Chloramphenicol | This study |
| pHAsgRNA*_farR_* | Knock out *farR* | Chloramphenicol | This study |
| pHAsgRNA*_argB_*_3_ | Mutate A26V, M31V and PAM of *argB* | Chloramphenicol | This study |
| pHAsgRNA*_argB_*_10_ | Mutate A26V, M31V, PAM and other 7 base pairs of *argB* | Chloramphenicol | This study |
| pHAsgRNA*_ldh_* | Knock out *ldh* | Chloramphenicol | This study |
| pHAsgRNA*_hdpA_* | Knock out *hdpA* | Chloramphenicol | This study |
| pHAsgRNA_ΔP_*_pgk_*_::P_*_hom_* | Replacement P*_pgk_* by P*_hom_* | Chloramphenicol | This study |
| pHAsgRNA_ΔP_*_pgk_*_::P_*_dapA_* | Replacement P*_pgk_* by P*_dapA_* | Chloramphenicol | This study |
| pOUT-*cas9-recET* | Knock out *cas9* and *recET* | Kanamycin | This study |

**Table S4** Primers used in the article.

| Primer | Sequence (5' - 3')^a^ | Note |  |
| --- | --- | --- | --- |
| P1 | *acagctatgacatgattacg*TTCCAACTGGCACGTTGTGT EcoRI | pIN-P*_hom_*-*cas9* |  |
| P2 | *tagttttcaacgg*CATGGGTAGAGCCTTTTGTTG | up_for_P*_hom_* (P1/P2) |  |
| P3 | *tctacccatg*CCGTTGAAAACTAAAAAGCTG |  |  |
| P4 | *attgagtatttcttatccat*GATTCTCCAAAAATAATCGC | P*_hom_* (P3/P4) |  |
| P5 | *gcgattatttttggagaatc*ATGGATAAGAAATACTCAAT |  |  |
| P6 | *tctcatccgccaaaacagcc*TCAGTCACCTCCTAGCTGAC | *cas9* (P5/P6) |  |
| P7 | *gtcagctaggaggtgactga*GGCTGTTTTGGCGGATGAGA |  |  |
| P8 | *tcttcctgtt*AGAGTTTGTAGAAACGCAAAAAGG | *rrnB* (P7/P8) |  |
| P9 | *tacaaactct*AACAGGAAGAGCCCGTAAAC |  |  |
| P10 | *taaaacgacggccagtgcca*TGGTCAAAGCTTCCCCTGGA HindIII | down (P9/P10) |  |
| P11 | GGGTCATCTCTGGCTGAATTGGAAGTCATGGGCGAACGCCGCATT | pXMJ19ts |  |
| P12 | CTTCCAATTCAGCCAGAGATGACCC | (P11/P12) |  |
| P13 | *caaagcatccggggctgatccccggcgcc*ATTCATGGAGAAACTGCG NarI | pHAsgRNA*_upp_* |  |
| P14 | *cccttagaaactt*AGCTTCACATGTTAAATCATTG | up_upp (P13/P14) |  |
| P15 | *aacatgtgaagct*AAGTTTCTAAGGGCATTACGG |  |  |
| P16 | *tagtggagtagct*TCCCATGATGAAGCAGCTC | down_upp (P15/P16) |  |
| P17 | *cttcatcatggga*AGCTACTCCACTAGTGTGATC |  |  |
| P18 | *cagccacccatcatcg*GCCCACAAGCATAGACCG | P*_glyA_* (P17/P18) |  |
| P19 | *ctatgcttgtgggccgatgatgggtggctgcttc*GTTTTAGAGCTAGAAATAGCAAG |  |  |
| P20 | *gctgaattcgagctcggtacccggggatcc*AAAAAAAGCACCGACTCG BamHI | tetra (P19/P20) |  |
| P21 | *cctatcaaacacgaaatctg*GCCCACAAGCATAGACCGCC | pHAsgRNA*_nc_*  up_upp (P13/P14)  down_upp (P15/P16)  P*_glyA_* (P17/P21) |  |
| P22 | *cagatttcgtgtttgatagg*GTTTTAGAGCTAGAAATAGC | tetra (P22/P20) |  |
| P23 | *taacggccaa*CATGGGTAGAGCCTTTTGTTG | pIN-P*_tuf_*-*cas9*  up_for_P*_tuf_* (P1/P23) |  |
| P24 | *tctacccatg*TTGGCCGTTACCCTGCGAAT |  |  |
| P25 | *attgagtatttcttatccat*TGTATGTCCTCCTGGACTTCG | P*_tuf_* (P24/P25) |  |
| P26 | *cgaagtccaggaggacataca*ATGGATAAGAAATACTCAAT | *cas9* (P26/P6)  *rrnB* (P7/P8)  down (P9/P10) |  |
| P27 | *cttgttgttgtatgtggggggtctgtatagct*TGTATGTCCTCCTGGACTTC | pIN-P*_tuf_*-RBS1-*cas9*  up_for_P*_tuf_* (P1/P23)  P*_tuf_* (P24/P27) |  |
| P28 | *accccccacatacaacaacaaggagacacgtt*ATGGATAAGAAATACTCAAT | *cas9* (P28/P6)  *rrnB* (P7/P8)  down (P9/P10) |  |
| P29 | *ttcttgttgtttgtagggggtctgtagtagct*TGTATGTCCTCCTGGACTTC | pIN-P*_tuf_*-RBS2-*cas9*  up_for_P*_tuf_* (P1/P23)  P*_tuf_* (P24/P29) |  |
| P30 | *agaccccctacaaacaacaagaaggagacggatt*ATGGATAAGAAATACTCAAT | *cas9* (P30/P6)  *rrnB* (P7/P8)  down (P9/P10) |  |
| P31 | *aggaaacagctatgacatgattacgaattc*GAACAAGCAGAAAATATTATTC EcoRI | pIN-P*_prp_*-*recET* |  |
| P32 | *gcctggcggtgta*CCGCTCCCGGCGGATTTG | cas_tail_*rrnB* (P31/P32) |  |
| P33 | *ccgccgggagcgg*TACACCGCCAGGCTGAATTATTC |  |  |
| P34 | *aaggaggaaacttt*ATGAGCACAAAACCACTCTTC | *recET*(anti) (P33/P34) |  |
| P35 | *gttttgtgctcat*AAAGTTTCCTCCTTTAAGCTTGCACACCACACTAATTCTTT |  |  |
| P36 | *ggctcttcctgtt*CTCCAGCGTCCAAGAATATG | P*_prp_*(anti) (P35/P36) |  |
| P37 | *ttggacgctggag*AACAGGAAGAGCCCGTAAAC |  |  |
| P38 | *cgttgtaaaacgacggccagtgccaagct*TGGTCAAAGCTTCCCCTG HindIII | down (P37/P38) |  |
| P39 | *tcataaagtttcctcctttaagcttccccctaatggccg*GCACACCACACTAATTCTTT | pIN-P*_prp_*-RBS1-*recET*  P*_prp_*(anti)-down (P39/P38) |  |
| P40 | *tcataaagtttcctcctttaagcttgtaatgttttggtc*GCACACCACACTAATTCTTT | pIN-P*_prp_*-RBS2-*recET*  P*_prp_*(anti)-down (P40/P38) |  |
| P41 | *CAAAGCATCCGGGGCTGATCCCCGGCGCC*AGATGCGCGTAATGCATGAG | pHA500sgRNA*_ldh_* |  |
| P42 | *TGGAGTTGCATAC*CCGCGCAATCCTGCAGAAC | up500 (P41/P42) |  |
| P43 | *CAGGATTGCGCGG*GTATGCAACTCCAACATC |  |  |
| P44 | *TAGTGGAGTAGCT*CACCACATTGCGATTTCC | down500 (P43/P44) |  |
| P45 | *TCGCAATGTGGTG*AGCTACTCCACTAGTGTGATC |  |  |
| P46 | *TGCGTAGGTCAGGATAT*GCCCACAAGCATAGACCG | P*_glyA_* (P45/P46) |  |
| P47 | *CTATGCTTGTGGGCATATCCTGACCTACGCAGTG*GTTTTAGAGCTAGAAATAGCAAG |  |  |
| P48 | *GCTGAATTCGAGCTCGGTACCCGGGGATCC*AAAAAAAGCACCGACTCG | tetra (P47/P48) |  |
| P49 | *CAAAGCATCCGGGGCTGATCCCCGGCGCC*GACTTCAATCGGCAGAGCG | pHA1000sgRNA*_ldh_* |  |
| P50 | *TGGAGTTGCATAC*CCGCGCAATCCTGCAGAAC | up1000 (P49/P50) |  |
| P51 | *CAGGATTGCGCGG*GTATGCAACTCCAACATCTCC |  |  |
| P52 | *TAGTGGAGTAGCT*ATACGACCACGGGCTACC | down1000 (P51/P52) |  |
| P53 | *CCCGTGGTCGTAT*AGCTACTCCACTAGTGTGATC |  |  |
| P54 | *TGCGTAGGTCAGGATAT*GCCCACAAGCATAGACCG | P*_glyA_* (P53/P54) |  |
| P55 | *CTATGCTTGTGGGCATATCCTGACCTACGCAGTG*GTTTTAGAGCTAGAAATAGCAAG |  |  |
| P56 | *GCTGAATTCGAGCTCGGTACCCGGGGATCC*AAAAAAAGCACCGACTCG | tetra (P55/P56) |  |
| P57 | *caaagcatccggggctgatccccggcgcc*TTCGATAATGTCATCGTGCTTCAAAAACC NarI | pHAsgRNA_ΔCGP3-1kb_ |  |
| P58 | *agacgatttcctc*TCTGCAGGCACTGCCTGG | upKO1kb (P57/P58) |  |
| P59 | *cagtgcctgcaga*GAGGAAATCGTCTCACGC |  |  |
| P60 | *tagtggagtagct*TCAGCGATGTCGCCAAAAG | downKO1kb (P59/P60) |  |
| P61 | *gcgacatcgctga*AGCTACTCCACTAGTGTG |  |  |
| P62 | *cgtaaacgagaatctcg*GCCCACAAGCATAGACCG | P*_glyA_* (P61/P62) |  |
| P63 | *ctatgcttgtgggccgagattctcgtttacggac*GTTTTAGAGCTAGAAATAGCAAG | tetra (P63/P20) |  |
| P64 | *caaagcatccggggctgatccccggcgcc*TGGTGTGGGTAAAACAGAAATG NarI | pHAsgRNA_ΔCGP3-10kb_ |  |
| P65 | *aatctttctggtg*GCTGGTAGAGATTGTCCAG | upKO10kb (P64/P65) |  |
| P66 | *aatctctaccagc*CACCAGAAAGATTAGCCTCATTATC |  |  |
| P67 | *tagtggagtagct*GATACGAGAAGAGCGGGTG | downKO10kb (P66/P67) |  |
| P68 | *ctcttctcgtatc*AGCTACTCCACTAGTGTG | P*_glyA_* (P68/P62)  tetra (P63/P20) |  |
| P69 | *caaagcatccggggctgatccccggcgcc*GTTTACCGCTTATTTACCTGGTC NarI | pHAsgRNA_ΔCGP3-20kb_ |  |
| P70 | *cctgatcttgacc*TTCGCCGATGCCGTAGATG | upKO20kb (P69/P70) |  |
| P71 | *cggcatcggcgaa*GGTCAAGATCAGGGTTATTAACCATTTTCTG |  |  |
| P72 | *tagtggagtagct*TACTCGTGCGCGTGTGGC | downKO20kb (P71/P72) |  |
| P73 | *acgcgcacgagta*AGCTACTCCACTAGTGTG | P*_glyA_* (P73/P62)  tetra (P63/P20) |  |
| P74 | *caaagcatccggggctgatccccggcgcc*TTCGTGTGTGGCTAGGCG NarI | p*gfp*HAsgRNA*_upp_* |  |
| P75 | *agggtaacggcca*TGGCTGCTGCACGGAAAG | up (P74/P75) |  |
| P76 | *cgtgcagcagcca*TGGCCGTTACCCTGCGAA |  |  |
| P77 | *ctcctttactcat*TGTATGTCCTCCTGGACTTCG | P*_tuf_* (P76/P77) |  |
| P78 | *aggaggacataca*ATGAGTAAAGGAGAAGAAC |  |  |
| P79 | *gactggctcatgg*TTATTTGTATAGTTCATCCATGCCATGTGTAATC | *gfp* (P78/P79) |  |
| P80 | *actatacaaataa*CCATGAGCCAGTCCCATAC |  |  |
| P81 | *tagtggagtagct*CTTCTTGGCTGAAGCCAC | down (P80/P81) |  |
| P82 | *ttcagccaagaag*AGCTACTCCACTAGTGTG | P*_glyA_* (P82/P18)  tetra (P19/P20) |  |
| P83 | *caaagcatccggggctgatccccggcgcc*TCTTAGGTATCATGGGTG NarI | p*gfp*HAsgRNA_CGP1_ |  |
| P84 | *agggtaacggcca*CCACGCTATTTTTTACAGTAAC | up (P83/P84) |  |
| P85 | *aaaaatagcgtgg*TGGCCGTTACCCTGCGAA | P*_tuf_* (P85/P77) |  |
| P86 | *tcgtcagattcta*TTATTTGTATAGTTCATCCATGCCATGTGTAATC | *gfp* (P78/P86) |  |
| P87 | *actatacaaataa*TAGAATCTGACGACGCATTC |  |  |
| P88 | *tagtggagtagct*TACCGAGTGGATTAGTTTC | down (P87/P88) |  |
| P89 | *aatccactcggta*AGCTACTCCACTAGTGTGATC |  |  |
| P90 | *tataaagagcactacaaag*GCCCACAAGCATAGACCG | P*_glyA_* (P89/P90) |  |
| P91 | *ctatgcttgtgggcctttgtagtgctctttatac*GTTTTAGAGCTAGAAATAGCAAG | tetra (P91/P20) |  |
| P92 | *caaagcatccggggctgatccccggcgcc*AGTCTTCTTCATCACCTTTC NarI | p*gfp*HAsgRNA_CGP2_ |  |
| P93 | *agggtaacggcca*ATCAGCGATATTTGATGAG | up (P92/P93) |  |
| P94 | *aaatatcgctgat*TGGCCGTTACCCTGCGAA | P*_tuf_* (P94/P77) |  |
| P95 | *gcatgcttgagtg*TTATTTGTATAGTTCATCCATGCCATGTGTAATC | *gfp* (P78/P95) |  |
| P96 | *actatacaaataa*CACTCAAGCATGCAAGCGTCAC |  |  |
| P97 | *tagtggagtagct*GCCCCCGTGAGGCTTCCT | down (P96/P97) |  |
| P98 | *gcctcacgggggc*AGCTACTCCACTAGTGTGATC |  |  |
| P99 | *cacagatcaatactggata*GCCCACAAGCATAGACCG | P*_glyA_* (P98/P99) |  |
| P100 | *ctatgcttgtgggctatccagtattgatctgtgg*GTTTTAGAGCTAGAAATAGCAAG | tetra (P100/P20) |  |
| P101 | *caaagcatccggggctgatccccggcgcc*TTTGCTTTCGATGAGTTC NarI | p*gfp*HAsgRNA_CGP3_ |  |
| P102 | *agggtaacggcca*GTGTGTAGTAGAAACCAATC | up (P101/P102) |  |
| P103 | *ttctactacacac*TGGCCGTTACCCTGCGAA | P*_tuf_* (P103/P77) |  |
| P104 | *gtcttgggtgatg*TTATTTGTATAGTTCATCCATGCCATGTGTAATC | *gfp* (P78/P104) |  |
| P105 | *actatacaaataa*CATCACCCAAGACTTAGGTTATC |  |  |
| P106 | *tagtggagtagct*GTTGATTGATGCCGCGATC | down (P105/P106) |  |
| P107 | *ggcatcaatcaac*AGCTACTCCACTAGTGTGATC | P*_glyA_* (P107/P62)  tetra (P63/P20) |  |
| P108 | *caaagcatccggggctgatccccggcgcc*GACTAATCCCGCTGCCTG NarI | p*hom-thrB*HAsgRNA*_upp_* |  |
| P109 | *agggtaacggcca*AGCTTCACATGTTAAATCATTGC | up (P108/P109) |  |
| P110 | *aacatgtgaagct*TGGCCGTTACCCTGCGAA |  |  |
| P111 | *cgtgtcttctagaaggc*TGTATGTCCTCCTGGACTTCG | P*_tuf_* (P110/P111) |  |
| P112 | *aggaggacatacagccttctagaagacacgtaaaaaaggaaaattaac*ATGACCTCAGCATCTGCC |  |  |
| P113 | *tttagtgtaattttgataaga*TTAGTCCCTTTCGAGGCG | *hom* (P112/P113) |  |
| P114 | *cgaaagggactaatcttatcaaaattacactaaaaagagaggtttcga*ATGGCAATTGAACTGAACG |  | |
| P115 | *cccttagaaactt*GCACTAATAAGGCCCCCTTC | *thrB* (P114/P115) |  |
| P116 | *gccttattagtgc*AAGTTTCTAAGGGCATTACGG |  |  |
| P117 | *tagtggagtagct*GGTGGGGCATGTGCATATC | down (P116/P117) |  |
| P118 | *cacatgccccacc*AGCTACTCCACTAGTGTGATC |  |  |
| P119 | *tgtgcatccggaatcat*GCCCACAAGCATAGACCG | P*_glyA_* (P118/P119) |  |
| P120 | *ctatgcttgtgggcatgattccggatgcacaggt*GTTTTAGAGCTAGAAATAGCAAG | tetra (P120/P20) |  |
| P192 | *caaagcatccggggctgatccccgg*ATATGCTCGATAGCCACG | p*lacZ*HAsgRNA*_cgl0900-cgl0901_*-_1_ |  |
| P193 | *agttgcagcaagc*GGGCAGGCCATAGAATCG | up (P192/P193) |  |
| P194 | *ctatggcctgccc*GCTTGCTGCAACTCTCTC |  |  |
| P195 | *tcggtcagaggct*CAGAAAGCAGACCAAACAG | *lacZ* fragment (P194/P195) |  |
| P196 | *ggtctgctttctg*AGCCTCTGACCGATGTCTC |  |  |
| P197 | *tagtggagtagct*CTGGTCAATCACCTGGGC | down (P196/P197) |  |
| P198 | *ggtgattgaccag*AGCTACTCCACTAGTGTGATC |  |  |
| P199 | *agcctctgaccgatgt*GCCCACAAGCATAGACCG | P*_glyA_* (P198/P199) |  |
| P200 | *ctatgcttgtgggcacatcggtcagaggctgggc*GTTTTAGAGCTAGAAATAGCAAG |  |  |
| P201 | *gctgaattcgagctcggtacccggg*AAAAAAAGCACCGACTCG | tetra (P200/P201) |  |
| P202 | *agttgcagcaagc*AATCGCGTGGCGGCGAGG | p*lacZ*HAsgRNA*_cgl0900-cgl0901_*-2  up(P192/P202) |  |
| P203 | *ccgccacgcgatt*GCTTGCTGCAACTCTCTC |  |  |
| P204 | *gggcaggccatag*CAGAAAGCAGACCAAACAG | *lacZ* fragment (P203/P204) |  |
| P205 | *ggtctgctttctg*CTATGGCCTGCCCAGCCT | down (P205/P197) |  |
| P206 | *gcgattctatggcctg*GCCCACAAGCATAGACCG | P*_glyA_* (P198/P206) |  |
| P207 | *ctatgcttgtgggccaggccatagaatcgcgtgg*GTTTTAGAGCTAGAAATAGCAAG | tetra (P207/P201) |  |
| P121 | *caaagcatccggggctgatccccggcgcc*TATGCACGCAGAGCCTTCG NarI | pHAsgRNA*_pgi1_* |  |
| P122 | CAATAAAGGAGTTTTC*g*TGGCGG | up*pgi*1 (P121/P122) |  |
| P123 | CCGCCACGAAAACTCCTTTATTG |  |  |
| P124 | *tagtggagtagct*TCCACCAGCGTTGCGTGA | down*pgi*1 (P123/P124) |  |
| P125 | *caacgctggtgga*AGCTACTCCACTAGTGTGATC |  |  |
| P126 | *catgaaaactcctttattg*GCCCACAAGCATAGACCG | P*_glyA_* (P125/P126) |  |
| P127 | *ccaataaaggagttttcatgg*GTTTTAGAGCTAGAAATAGCAAG | tetra (P127/P20) |  |
| P128 | CAATAAAGGAGTTTTCGTGGCAGACATTTCG | pHAsgRNA*_pgi2_*  up*pgi*2 (P121/P128) |  |
| P129 | CGAAATGTCTGCCACGAAAACTCCTTTATTG | down*pgi*2 (P129/P124)  P*_glyA_* (P125/P126)  tetra (P127/P20) |  |
| P130 | *caaagcatccggggctgatccccggcgcc*TTTCCATCATCGCTCCTG NarI | pHAsgRNA*_argR_* |  |
| P131 | *taattggcagcta*GGGATTTAAGTTTTCCGG | *argR*_up (P130/P131) |  |
| P132 | *aaacttaaatccc*TAGCTGCCAATTATTCCG |  |  |
| P133 | *tgcggttagtcat*GGGTAAAAAATCCTTTCGTAG | P*_sod_* (P132/P133) |  |
| P134 | *ggattttttaccc*ATGACTAACCGCATCGTTC |  |  |
| P135 | *tagtggagtagct*CTCCAGGTTTGGATCGGTG | *argG* (P134/P135) |  |
| P136 | *tccaaacctggag*AGCTACTCCACTAGTGTGATC |  |  |
| P137 | *atgatctgtagaaaccag*GCCCACAAGCATAGACCG | P*_glyA_* (P136/P137) |  |
| P138 | *ctatgcttgtgggcctggtttctacagatcattc*GTTTTAGAGCTAGAAATAGCAAG | tetra (P138/P20) |  |
| P139 | *caaagcatccggggctgatccccggcgcc*CTTGGCTTTGAGTTCGTTGGAACC NarI | pHAsgRNA*_farR_* |  |
| P140 | *cgggcttatcgcc*CCACGAGAACGCGGTCGG | *farR*_up (P139/P140) |  |
| P141 | *cgcgttctcgtgg*GGCGATAAGCCCGTGGAA |  |  |
| P142 | *tagtggagtagct*AAGGTAGGAGATTGGCAGAG | *farR*_down (P141/P142) |  |
| P143 | *aatctcctacctt*AGCTACTCCACTAGTGTGATC |  |  |
| P144 | *gcgaccagatcaccaat*GCCCACAAGCATAGACCG | P*_glyA_* (P143/P144) |  |
| P145 | *ctatgcttgtgggcattggtgatctggtcgcgtc*GTTTTAGAGCTAGAAATAGCAAG | tetra (P145/P20) |  |
| P146 | *caaagcatccggggctgatccccggcgcc*GCTGTGACAGTGGTGGGAAC NarI | pHAsgRNA*_argB3_* |  |
| P147 | TTGCGCCCACTGTACGTAAGAAAACTACATCAGCAGCAAAGACAGCCTTGA | up_*argB* (P146/P147) |  |
| P148 | GGTGCGCAAGAAGACCACGTCAGCAGCAAAAACAGCCTTG |  |  |
| P149 | CAAGGCTGTTTTTGCTGCTGACGTGGTCTTCTTGCGCACC | down_*argB* (P148/P149) |  |
| P150 | *gattgctccaggc*AGCTACTCCACTAGTGTGATC |  |  |
| P151 | *ggtcttcttgcgcacc*GCCCACAAGCATAGACCG | P*_glyA_* (P150/P151) |  |
| P152 | *ctatgcttgtgggcggtgcgcaagaagaccatgt*GTTTTAGAGCTAGAAATAGCAAG | tetra (P152/P20) |  |
| P153 | TCAAGGCTGTcTTTGCTGCtGAtGTaGTtTTCTTaCGtACaGTGGGCGCAA | pHAsgRNA*_argB10_* |  |
| P154 | *tagtggagtagct*GCCTGGAGCAATCGTAGAG | up_*argB* (P146/P147)  down_*argB* (P153/P154)  P*_glyA_* (P150/P151)  tetra (P152/P20) |  |
| P155 | *CAAAGCATCCGGGGCTGATCCCCGGCGCC*GAGAATTTCGGCGTGCTC NarI | pHAsgRNA*ldh* |  |
| P156 | *AGTGGGATCGAAA*ATCTTTGGCGCCTAGTTG | up_*ldh* (P155/P156) |  |
| P157 | *AGGCGCCAAAGAT*TTTCGATCCCACTTCCTG |  |  |
| P158 | *TAGTGGAGTAGCT*AAAACAGCCAGGTTAGCAG | down_*ldh* (P157/P158) |  |
| P159 | *ACCTGGCTGTTTT*AGCTACTCCACTAGTGTGATC |  |  |
| P160 | *ACAACACCATGGTTTAAG*GCCCACAAGCATAGACCG | P*_glyA_* (P159/P160) |  |
| P161 | *CTATGCTTGTGGGCCTTAAACCATGGTGTTGTGT*GTTTTAGAGCTAGAAATAGCAAG | tetra (P161/P20) |  |
| P162 | *CAAAGCATCCGGGGCTGATCCCCGGCGCC*TTTAGACCCGGGGTACGG NarI | pHAsgRNA*_hdpA_* |  |
| P163 | *GACCTACAGAATA*AACACCATTGTCCCTGTTTTG | up_*hdpA* (P162/P163) |  |
| P164 | *GGACAATGGTGTT*TATTCTGTAGGTCATGGCATTTGCAG |  |  |
| P165 | *TAGTGGAGTAGCT*CTCGGGGAAGCCAGGTGA | down_*hdpA* (P164/P165) |  |
| P166 | *TGGCTTCCCCGAG*AGCTACTCCACTAGTGTGATC |  |  |
| P167 | *TCACGTGATCAATTCCAT*GCCCACAAGCATAGACCG | P*_glyA_* (P166/P167) |  |
| P168 | *CTATGCTTGTGGGCATGGAATTGATCACGTGAGT*GTTTTAGAGCTAGAAATAGCAAG | tetra (P168/P20) |  |
| P169 | *CAAAGCATCCGGGGCTGATCCCCGGCG*CCTCATGACCACCGTTCAC NarI | pHAsgRNA*_Phom_* |  |
| P170 | *TAGTTTTCAACGG*TAGCGATGTGAACTAATTAGAGC | up_P*_pgk_* (P169/P170) |  |
| P171 | *GTTCACATCGCTA*CCGTTGAAAACTAAAAAGC |  |  |
| P172 | *TCTTAACAGCCAT*GATTCTCCAAAAATAATCGC | P*_hom_* (P171/P172) |  |
| P173 | *TTTTTGGAGAATC*ATGGCTGTTAAGACCCTCAAGGACTTG |  |  |
| P174 | *TAGTGGAGTAGCT*ACGGAGGTCTGTGCGCGG | down_P*_pgk_* (P173/P174) |  |
| P175 | *CACAGACCTCCGT*AGCTACTCCACTAGTGTGATC |  |  |
| P176 | *CGGGCTATTGGGATGACACA*GCCCACAAGCATAGACCG | P*_glyA_* (P175/P176) |  |
| P177 | *CTATGCTTGTGGGCTGTGTCATCCCAATAGCCCG*GTTTTAGAGCTAGAAATAGCAAG | tetra (P177/P20) |  |
| P178 | *TGTGAGCTTTGCG*TAGCGATGTGAACTAATTAGAGC | pHAsgRNA_P_*_dapA_*  up_P*_pgk_* (P169/P178) |  |
| P179 | *GTTCACATCGCTA*CGCAAAGCTCACACCCAC |  |  |
| P180 | *TCTTAACAGCCAT*AGAGTTCAAGGTTACCTTCTTCC | P*_dapA_* (P179/P180) |  |
| P181 | *AACCTTGAACTCT*ATGGCTGTTAAGACCCTCAAGGACTTG | down_P*_pgk_* (P181/P174)  PglyA (P175/P176)  tetra (P177/P20) |  |
| P182 | *TTCACACAGGAAACAGAATTAATTAAGCTTGCCCTCGTAAGCTAGAGGAGGTTAAC*ATGGAACTGACGACTCGC HindIII | pXMJ19-*mgsA-gldA-yqhD* |  |
| P183 | *ACCTCCTTTCTGTTTGTTGTCCTTGCGGAATAGCG*TTACTTCAGACGGTCCGC | *mgsA* (P182/P183) |  |
| P184 | *TTCCGCAAGGACAACAAACAGAAAGGAGGTAAAAC*ATGGACCGCATTATTCAATC |  |  |
| P185 | *ACCTCCTCTTTGTATTTCTGTGTACTGG*TTATTCCCACTCTTGCAG | *gldA* (P184/P185) |  |
| P186 | *CCAGTACACAGAAATACAAAGAGGAGGTAACTA*ATGAACAACTTTAATCTGCAC |  |  |
| P187 | *GCTGAATTCGAGCTCGGTACCCGGGGATCC*TTAGCGGGCGGCTTCGTATATAC BamHI | *yqhD* (P186/P187) |  |
| P188 | *AGGAAACAGCTATGACATGATTACGA*ATTCCAGCGTCACGACGTTC | pOUT-*cas9*-*recET* |  |
| P189 | *GGCTCTTCCTGTT*CATGGGTAGAGCCTTTTGTTG | up (P188/P189) |  |
| P190 | *GGCTCTACCCATG*AACAGGAAGAGCCCGTAAAC |  |  |
| P191 | *CGTTGTAAAACGACGGCCAGTGCCAAGCT*TGGGAAGACTCGCCTCTG | down (P190/P191) |  |
| Pv1 | GTGGTAAATCGGATAACGTT | Verfication of integration of *cas9* |  |
| Pv2 | TTGGCATCTGCTAAAATAA |  |  |
| Pv3 | GCTTCGGCAATCATCAGTC | Verification of deletion at *upp* |  |
| Pv4 | TGGGTATTTTGCGTCCTC |  |  |
| Pv5 | AAAAGGAAATGAGCTGGCTCTGC | Amplification of *cas9* region in RT-PCR |  |
| Pv6 | TGCTTATGCTGCTCCACAAACAA |  |  |
| Pv7 | CGTACTGCTGAAGGCTCTT | Amplification of rpoB region in RT-PCR |  |
| Pv8 | TTTGCTACACCATCGGACT |  |  |
| Pv58 | GGAGGGTGCCGTTGGAGATGTAG | Verification of deletion of *ldh* |  |
| Pv59 | CCGGTGCCCGTGGAATTGTTGAT |  |  |
| Pv9 | CTTCTACCGTCTTCGTCAGT | Verification of 1-kb deletion at CGP3 |  |
| Pv10 | TTGTGCTGGTTGGGTTTAAT |  |  |
| Pv11 | ACTGTGACTATTAGGGGTGG |  |  |
| Pv12 | AAGTGCCAGCCTCCATCAAT | Verification of 10-kb deletion at CGP3 |  |
| Pv13 | TTAGCGGCTGTTGTGCCGGC |  |  |
| Pv14 | AAGGATGCGATGTGGTCTTC |  |  |
| Pv15 | GCTCTCCGCCCATTATGAGC | Verification of 20-kb deletion at CGP3 |  |
| Pv16 | AGCAGGCACAGGATGCATAT |  |  |
| Pv17 | GGTTTGCTAGTGCATAGTCATTT |  |  |
| Pv18 | GAATCAAATGGTGAGTTCG | Verification of editing at CGP1 locus |  |
| Pv19 | CTCTGACCCTCGCTAGTTG |  |  |
| Pv20 | TTTCCGAAGAAGACTTAGAACCT | Verification of editing at CGP2 locus |  |
| Pv21 | TCTGTGAATACTTTAATCCGCTC |  |  |
| Pv22 | AATGTTGAGGCAGCCCTACAGAT | Verification of editing at CGP3 locus |  |
| Pv23 | TCAATCATGGTGGTGTCGTCTTT |  |  |
| Pv62 | GCAATCATCAGTCCAGGAAG | Verification of insertions of 2.5-kb, 5.7-kb and 7.5-kb fragments |  |
| Pv63 | CGCATCCTCAGCAAGAACTC |  |  |
| Pv64 | AGGAGTGCGTGTCAGACGAATG | Verification of insertions of the *lacZ* fragment |  |
| Pv65 | AGCCAGATTCGCAAGGGTC |  |  |
| Pv26 | CCTACATGATTGGCTTTGC | Verification of editing at *argR* locus |  |
| Pv27 | GACTTGATGCCCACGAGA |  |  |
| Pv28 | CTCCAGGACGACGAAATCA | Verification of editing at *farR* locus |  |
| Pv29 | AATCAACTTGGCGGAACTCT |  |  |
| Pv30 | CTGATGCAGAGGGTGTGACCA | Verification of editing at *argB* locus |  |
| Pv31 | TTAATGTTGTAAATCTGGC |  |  |
| Pv32 | CGCGACCTACTTCTGCGCCTGGG | Verification of editing at *ldh* locus |  |
| Pv33 | CCCGCGATCGTCTCCTTCGGTCC |  |  |
| Pv34 | TGGGTAAAAGGTTTGTAGGTAGTCG | Verification of editing at *hdpA* locus |  |
| Pv35 | ACTCTTGGAGGGTTTCAAAGGTG |  |  |
| Pv36 | CATCATCTCCGCACCAGCAAGCA | Verification of editing at P*_pgk_* locus |  |
| Pv37 | GCAGCCAGCGCCTCAATAACACC |  |  |
| Pv38 | ACTTGCAGACGACGCACTTCCAT | Amplification of *fda* region in RT-PCR |  |
| Pv39 | GAACGCCGTTGTAGTTCTCAAACA |  |  |
| Pv40 | GTGTCCCTGCACTCTGACTTGCG | Amplification of *gapX* region in RT-PCR |  |
| Pv41 | GGTTGGAGTAGCCGAACTCGTTGT |  |  |
| Pv42 | GCATCCGAATTTAACAAGGACGCAGAG | Amplification of *gpk* region in RT-PCR |  |
| Pv43 | CCCATTGGGCCGTTCCAGAAGAT |  |  |
| Pv44 | ATGCCTCAAGGACGTTGTGGTTC | Amplification of *gpmA* region in RT-PCR |  |
| Pv45 | CAGTGGGATGCCGGTTGGGATGT |  |  |
| Pv46 | CGCCTGAAGGAGGGCATCGCTAA | Amplification of *eno* region in RT-PCR |  |
| Pv47 | CAATGGTGGTGTCCTCGGTCTCA |  |  |
| Pv48 | GCCAACACCGTTATCGCGTATGA | Amplification of *tpiA* region in RT-PCR |  |
| Pv49 | GCCTTAACAGAACCACCGTAAAGAAT |  |  |
| Pv50 | CTTCGGTTCCAAGGTTCCAGGTT | Amplification of *zwf* region in RT-PCR |  |
| Pv51 | CCAGCTCAGTTCCACTTCCTCGTT |  |  |
| Pv52 | TCTGGAAGGCACCAAGGAGAAGG | Amplification of *tkt* region in RT-PCR |  |
| Pv53 | CCATGCAAGGAACGGAAACAACG |  |  |
| Pv54 | TTCGTATGATCGGTTCCGCACAGGC | Amplification of *gltA* region in RT-PCR |  |
| Pv55 | GTCGCCACCGTGGTTGCTCTTGATG |  |  |
| Pv56 | ACCTACACCGACGACGCTGTTTCCG | Amplification of *acn* region in RT-PCR |  |
| Pv57 | GTTGTCAGCTTCGACGCCGCCTTCA |  |  |
| Pv60 | CGCATCCAGCTCAATT | Verification of the deletion of *cas9* and *recET* |  |
| Pv61 | CAATCCGTCAAGTCCC |  |  |

1. Italic sequences represent the overlapping sequences for Gibson Assembly [9]. Underlined sequences represent the corresponding enzymes for linearizing the backbone plasmids.

**SUPPLEMENTARY REFERENCES**

1. Jiang Y, Qian F, Yang J, Liu Y, Dong F, Xu C, Sun B, Chen B, Xu X, Li Y, et al: CRISPR-Cpf1 assisted genome editing of Corynebacterium glutamicum**.** Nat Commun 2017, 8**:**15179.

2. Cho JS, Choi KR, Prabowo CPS, Shin JH, Yang D, Jang J, Lee SY: CRISPR/Cas9-coupled recombineering for metabolic engineering of Corynebacterium glutamicum**.** Metab Eng 2017, 42**:**157-167.

3. Peng F, Wang X, Sun Y, Dong G, Yang Y, Liu X, Bai Z: Efficient gene editing in Corynebacterium glutamicum using the CRISPR/Cas9 system**.** Microb Cell Fact 2017, 16**:**201.

4. Liu J, Wang Y, Lu Y, Zheng P, Sun J, Ma Y: Development of a CRISPR/Cas9 genome editing toolbox for Corynebacterium glutamicum**.** Microb Cell Fact 2017, 16**:**205.

5. Schafer A, Tauch A, Jager W, Kalinowski J, Thierbach G, Puhler A: Small mobilizable multi-purpose cloning vectors derived from the Escherichia coli plasmids pK18 and pK19: selection of defined deletions in the chromosome of Corynebacterium glutamicum**.** Gene 1994, 145**:**69-73.

6. Zhang G, Wang W, Deng A, Sun Z, Zhang Y, Liang Y, Che Y, Wen T: A mimicking-of-DNA-methylation-patterns pipeline for overcoming the restriction barrier of bacteria**.** PLoS Genet 2012, 8**:**e1002987.

7. Jakoby M, Ngouoto-Nkili CE, Burkovski A: Construction and application of new Corynebacterium glutamicum vectors**.** Biotechnol Tech 1999, 13**:**437-441.

8. Jiang Y, Chen B, Duan C, Sun B, Yang J, Yang S: Multigene editing in the Escherichia coli genome via the CRISPR-Cas9 system**.** Appl Environ Microbiol 2015, 81**:**2506-2514.

9. Gibson DG, Young L, Chuang RY, Venter JC, Hutchison CA, 3rd, Smith HO: Enzymatic assembly of DNA molecules up to several hundred kilobases**.** Nat Methods 2009, 6**:**343-345.
