# Supplementary material for: A RecET-assisted CRISPR–Cas9 genome editing in Corynebacterium glutamicum
Source: Microb Cell Fact. 2018 Apr 23;17:63. doi: 10.1186/s12934-018-0910-2 (PMC5913818; doi:10.1186/s12934-018-0910-2)
Supplement: Supplementary file 2 — Additional file 2: Standard protocol of a RecET-assisted CRISPR-Cas9 genome editing in Corynebacterium glutamicum. [file 12934_2018_910_MOESM2_ESM.docx]

**Standard protocol of a RecET-assisted CRISPR-Cas9 genome editing in *Corynebacterium glutamicum***

**Step 1: Plug in Cas9 and RecET expression cassettes into chromosome**

To introduce *cas9* expression cassette into the chromosome, *cas9* gene was amplified using pCas as the template with primers P30/P6. P*_tuf_*, the upstream homologous arms (HA), and downstream HA were amplified using *C. glutamicum* ATCC 13032 genome as the template with primers P24/P29, P1/P23 and P9/P10, respectively. The terminator *rrnB* was amplified from pXMJ19 with primers P7/P8. These fragments together with linearized pK18*mobsacB* digested by *Eco*RI and *Hind*III were purified and recovered for Gibson assembly with NEBuilder Master Mix (NEB) to construct pIN-P*_tuf_*-rbs2-*cas9*. The resultant plasmid is transformed into competent cells of *C. glutamicum* by electroporation. The cells are recovered in 1 mL BHIS medium at 30 °C for 2 hours. Then cells are cultivated on the BHIS plate containing 25 mg/L kanamycin at 30 °C for 2 days. The transformants were picked and grown at LB plate supplemented with 20% sucrose. The colonies are further tested for kanamycin resistance. The kanamycin-sensitive colonies are identified by colony PCR with DNA sequencing to obtain the WT::P*_tuf_*-rbs2-*cas9* strain.

To integrate RecET expression cassette into the chromosome, *E. coli* W3110 genome was used as a template to amplify *recET*(anti) with primers P33/P34. WT::P*_tuf_*-Cas9 genome was used as a template to amplify *cas*_tail_*rrnB* with primers P31/P32. WT::P*_tuf_*-Cas9 genome was used as a template to amplify P*_prp_*(anti) and downstream HA with primers P35/P36 and P37/P38, respectively. These fragments together with linearized pK18*mobsacB* digested by *Eco*RI and *Hind*III were purified and recovered for Gibson assembly to construct the plasmid pIN-P*_prp_*-*recET* harboring *recET* under the control of P*_prp_* promoter. To increase the translation initiation rate of RecET, pIN-P*_prp_*-*recET* was used as a template to amplify P*_prp_*(anti)-down with primers P39/P38 to introduce RBS4 to *recET*. The fragment with linearized pIN-P*_prp_*-*recET* digested by HindIII were purified and recovered for Gibson assembly to construct the plasmid pIN-P*_prp_*-rbs4-*recET*. Then, the resultant plasmid were transformed to WT::P*_tuf_*-rbs2-*cas9* by electroporation. Screening for the ﬁrst and second recombination events were performed as above described to obtain WT::P*_tuf_*-rbs2-*cas9*::P*_prp_*-rbs4-*recET* strain (referred as EDT).

**Step 2: Assembly of sgRNA and donor DNA parts to construct sgRNA expression plasmid**

The donor DNA containing the upstream and downstream homologous arms (HAs) for gene deletion or site mutation were amplified from genomic DNA. For insertion, the inserted gene was ligated to the middle of two HAs by overlap extension PCR. The N20 targeting each specific site in the genome was designed using the online tools (www.rgenome.net/cas-designer) and synthesized in primers. The designed N20 was inserted between P*_glyA_* promoter amplified by PCR using *C. glutamicum* genome as the template and the gRNA scaffold amplified by PCR using pTargetF as the template. The linearized pXMJ19ts, homologous arms, P*_glyA_* and gRNA scaffold were assembled to construct pHAsgRNA plasmid.

**Step 3: Procedure for genome editing**

100 ng of the pHAsgRNA plasmid was firstly transformed by electroporation into the competent cells of EDT. Then cells were recovered in the BHIS medium supplementing with 0.5 g/L sodium propionate (PRP) for RecET induction at 30 °C for 2 hours. After that, cells were spread on BHIS plate containing 10 μg/ml chloramphenicol for 2 days. The transformants were screened by colony PCR with corresponding primers and verified by DNA sequencing to confirm the chromosomal deletion or insertion of target genes.

**Step 4: Plasmid curing for the successive round of genome editing**

The positive transformants were incubated in LB medium at 37 °C for overnight cultivation. The cultures were diluted by 1000 folds and plated to LB plates. The obtained colonies were tested for chloramphenicol sensitivity. The chloramphenicol-sensitive colonies were ready for the next round of genetic manipulation. And then, new editing pHAsgRNA plasmids were transformed by electroporation for iterative genome editing.

**Step 5: Plug out Cas9 and RecET expression cassettes from the chromosome**

The upstream and downstream HAs of chromosome-borne *cas9-recET* expression cassettes were amplified using P188/P189 and P190/P191, and then ligated into the pK18*mobsacB* by Gibson Assembly to construct pOUT-*cas9*-*recET*. Subsequently, the pOUT-*cas9*-*recET* plasmid was transformed into the edited strains by electroporation to delete *cas9-recET* expression cassettes by two rounds of homologous recombination as described in step 1.
